# Pathogen induced subversion of NAD^+^ metabolism mediating host cell death: a target for development of chemotherapeutics

**DOI:** 10.1038/s41420-020-00366-z

**Published:** 2021-01-13

**Authors:** Ayushi Chaurasiya, Swati Garg, Ashish Khanna, Chintam Narayana, Ved Prakash Dwivedi, Nishant Joshi, Zill e Anam, Niharika Singh, Jhalak Singhal, Shikha Kaushik, Amandeep Kaur Kahlon, Pallavi Srivastava, Manisha Marothia, Mukesh Kumar, Santosh Kumar, Geeta Kumari, Akshay Munjal, Sonal Gupta, Preeti Singh, Soumya Pati, Gobardhan Das, Ram Sagar, Anand Ranganathan, Shailja Singh

**Affiliations:** 1 Special Centre for Molecular Medicine, Jawaharlal Nehru University, New Delhi, 110067 India; 2 Department of Chemistry, Institute of Science, Banaras Hindu University, Varanasi, 221005 Uttar Pradesh India; 3 International Centre for Genetic Engineering and Biotechnology, New Delhi, 110067 India; 4 Department of Life Sciences, School of Natural Sciences, Shiv Nadar University, Greater Noida, 201314 India

**Keywords:** Antiparasitic agents, Mechanisms of disease

## Abstract

Hijacking of host metabolic status by a pathogen for its regulated dissemination from the host is prerequisite for the propagation of infection. *M. tuberculosis* secretes an NAD^+^-glycohydrolase, TNT, to induce host necroptosis by hydrolyzing Nicotinamide adenine dinucleotide (NAD^+^). Herein, we expressed TNT in macrophages and erythrocytes; the host cells for *M. tuberculosis* and the malaria parasite respectively, and found that it reduced the NAD^+^ levels and thereby induced necroptosis and eryptosis resulting in premature dissemination of pathogen. Targeting TNT in *M. tuberculosis* or induced eryptosis in malaria parasite interferes with pathogen dissemination and reduction in the propagation of infection. Building upon our discovery that inhibition of pathogen-mediated host NAD^+^ modulation is a way forward for regulation of infection, we synthesized and screened some novel compounds that showed inhibition of NAD^+^-glycohydrolase activity and pathogen infection in the nanomolar range. Overall this study highlights the fundamental importance of pathogen-mediated modulation of host NAD^+^ homeostasis for its infection propagation and novel inhibitors as leads for host-targeted therapeutics.

## Introduction

Intracellular pathogens have evolved strategies to manipulate host cell pathways for dissemination and infection propagation. Unlike independent organisms, intracellular pathogens depend on the activities of host cell to complete their life cycle. Some of these pathogens have evolved strategies for host manipulation to increase their survival causing detrimental effects to the host, leading to its death. Because dissemination is an essential aspect of pathogenesis, targeting or modulating host processes involved in pathogenesis can control the infection^1^. Pathogen-mediated changes in host intracellular nicotinamide adenine dinucleotide (NAD^+^), an essential coenzyme and a redox factor regulating numerous cellular metabolic pathways, levels have been observed in various diseases^2,3^. In particular, host uses NAD^+^ as a substrate for a group of “NAD^+^-dependent” enzymes^4,5^.

Recently, *Mycobacterium tuberculosis*, the causative agent for tuberculosis, mediated modulation of host NAD^+^ homeostasis has been presented as one of the most fascinating examples of a pathogen’s infection strategy wherein NAD^+^ depletion through TNT activates necroptosis pathways in order to facilitate growth and spread of *M. tuberculosis*^6–8^. However, the regulation of NAD^+^ metabolism in pathogen dissemination and its utilization as a potential drug target are areas as yet unexplored. One of the best examples of NAD^+^ dependent enzymes are NAD^+^ glycohydrolase^9^. These NADases are identified as virulence factors in *Streptococcus pyogenes*^10,11^, and in *M. tuberculosis*^6–8^. Upon infection *M. tuberculosis* secretes a protein called TNT (Tuberculosis necrotizing toxin) into the host cytosol^6^ wherein it plays a major role in *M. tuberculosis* pathogenesis as its β-NAD^+^ glycohydrolase activity depletes cellular NAD^+^ pool and leads to host cell death by necroptosis^7,8^. To protect itself from the toxic effect of TNT, an endogenous protein of *M. tuberculosis* called IFT (Immunity factor for TNT) forms a complex with TNT and acts as the antitoxin for TNT^7,12^. Host cell death allows the intracellular pathogen to evade immune responses and disseminate^13,14^, and therefore host-directed therapies to prevent NAD^+^ depletion could help treat tuberculosis through inhibition of necroptosis.

Several lines of evidence indicate a role for NAD^+^ in the pathology of malaria, one of the most devastating and prevalent infectious diseases with 228 million cases and over 0.4 million deaths in 2018^15–18^. *Plasmodium spp*. is the causative agent of malaria and it has previously been shown that *P. falciparum*-infected erythrocytes have higher NAD^+^ levels compared to uninfected erythrocytes^16^ and that these elevated NAD^+^ levels might aid *P. falciparum* in establishing infection^17^. Malaria parasite hijacks a number of host cell proteins and pathways for the progression of its intra-erythrocytic stage. For example, the parasite exploits host calpain and tyrosine kinase to facilitate its egress from infected erythrocyte^19,20^. Furthermore, *P. falciparum* largely depends on the host for its supply of lipids and fatty acids^21,22^. However, the role of host erythrocyte NAD^+^ in supporting parasite infection remain unexplored till date.

The role of intra-erythrocytic NAD^+^ levels in inducing erythrocyte death, known as eryptosis, remain unknown. Eryptosis is elicited by various stimulators including oxidative stress, energy depletion, osmotic shock and specific xenobiotics^23–25^. There exists the possibility that NAD^+^ depletion is able to elicit eryptosis in erythrocytes in the way similar to how it triggers necrosis in other cases^8,10,26^, although the precise molecular mechanism behind this might be distinctive given that erythrocytes are enucleated cells. The link between these two mechanisms, eryptosis and NAD^+^ modulation, is largely unknown and role of NAD^+^ in *P. falciparum* infection is also an open question. The present study explores the potential link between NAD^+^ modulation, eryptosis and parasite infection.

In this study, we show targeting TNT through IFT allows survival of the macrophage thereby further limiting *M. tuberculosis* growth. Next, TNT was used to modulate the intra-erythrocytic NAD^+^ levels that stimulates premature eryptosis and further reduces parasite propagation showing importance of NAD^+^ in *P. falciparum* infection. Finally, we demonstrate that two lead compounds, **8** and **9**, display no toxicity towards host cells but are effective in blocking NAD^+^-glycohydrolase activity of TNT, showing potent inhibitory potential against both *M. tuberculosis* and *P. falciparum*. This study establishes the role of NAD^+^ metabolism in pathogen propagation and paves the path for drug-intervention for the treatment of malaria, tuberculosis, and other diseases where NAD^+^ modulation is a pathological factor.

## Results

### Endogenous NAD^+^ levels determines bacterial colony formation and propagation

**Fig. 1:**
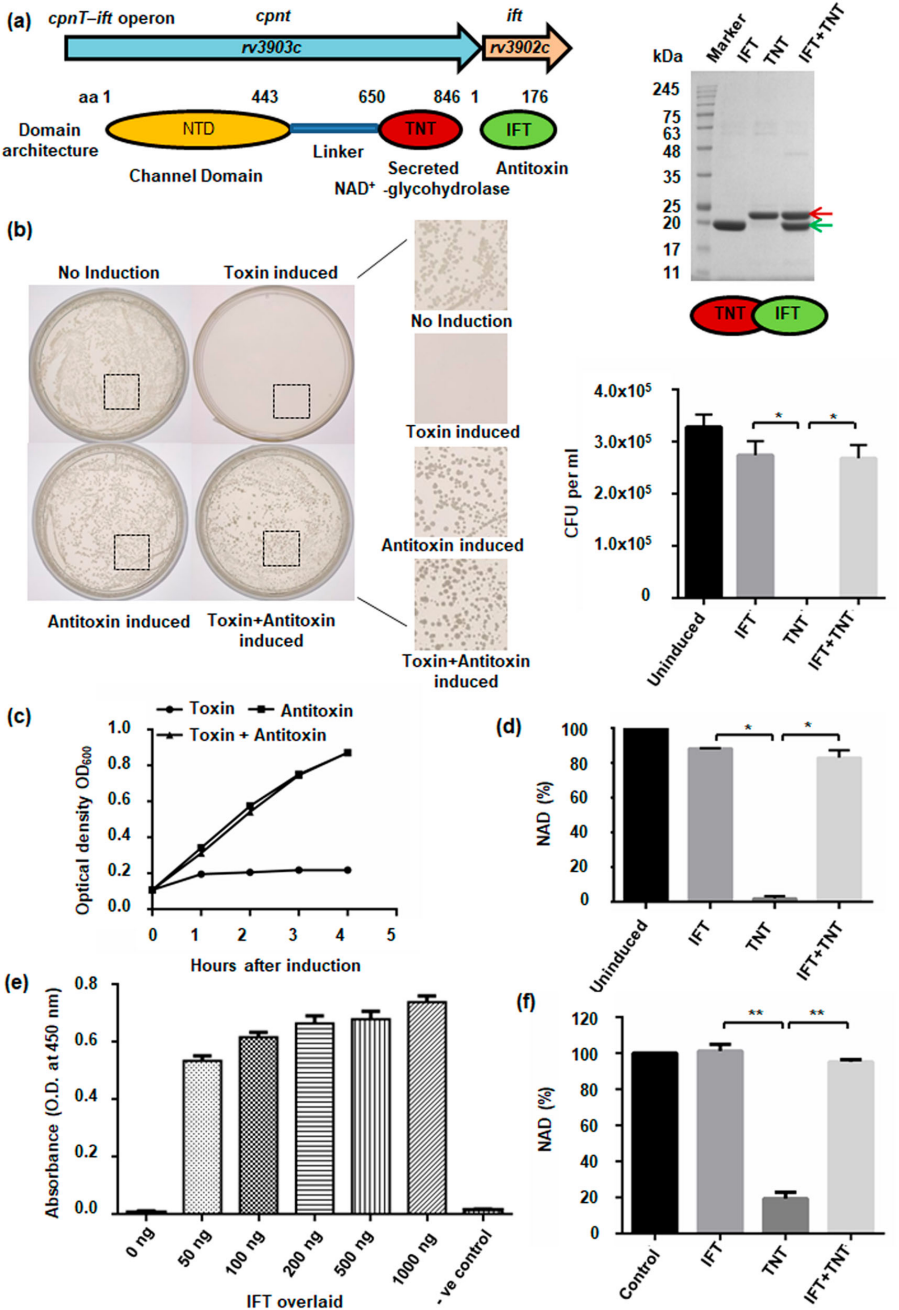
Endogenous NAD^+^ levels determine bacterial colony formation and propagation. **a** Schematic representation of the *cpnT–ift* operon of *M. tuberculosis* along with domain architecture of proteins Cpnt and IFT, and SDS-PAGE analysis of purified recombinant proteins, IFT-His_6x_ (green arrow, molecular weight ~21.6 kDa) and TNT-His_6x_ (red arrow, molecular weight ~24.6 kDa). *E. coli* BL21 (DE3) cells were co-transformed with IFT-pET28a and TNT-pMTSA plasmids. TNT expression was induced with arabinose while IPTG was used for the overexpression of IFT. **b** Growth of co-transformed cells on agar plates applied with 0.2% arabinose, or 25 µM IPTG, or both after 16 h of incubation at 37 °C. Control represents growth on agar plate containing antibiotics only. Number of bacterial colonies on agar plates, containing streptomycin and kanamycin supplemented with 0.2% arabinose or 25 µM IPTG or both, were counted. **c** Optical density at 600 nm was measured in culture induced with arabinose (0.2%) or 1 mM IPTG or both at time zero as well as different time points after that. The result represents mean ± SD for an experiment carried out in triplicate. **d** Intracellular NAD^+^ levels of bacterial cells expressing rIFT or rTNT, or both, in comparison to control cells, was determined by an enzyme-coupling assay. **e** ELISA based confirmation of the interaction between IFT and TNT. Purified recombinant TNT (200 ng) was coated on ELISA plates and overlaid with different amounts of IFT or buffer alone as control and subsequently detected by anti-IFT antibody followed by HRP conjugated secondary anti-mouse antibody. Data represents mean ± SD for experiments carried out in triplicate. **f** NAD^+^-glycohydrolase assay of purified rTNT (75 nM) at 200 µM NAD^+^ concentration in the presence and absence of IFT by the NADH fluorescence method. rIFT inhibits the NAD^+^-glycohydrolase activity of rTNT. Reaction without rTNT was used as control. NAD^+^ concentration in the control sample was considered as 100% and other samples were normalized compared to the untreated control sample. *P* values were calculated using unpaired *t*-test with Welch correction. *P < 0.05, **P < 0.01, ***P < 0.001. Statistical significance was determined using TNT-treated sample as the control against which other groups were compared.

The *M. tuberculosis* outer membrane protein CpnT (channel protein with necrosis-inducing toxin) consists of an N-terminal outer membrane channel domain and a secreted C-terminal TNT^6,7^. *M. tuberculosis* also produces IFT, a natural inhibitor of TNT^7,12^ (Fig. 1a, Supplementary Fig. 1, Supplementary Table 1). To study the effect of endogenous NAD^+^ levels on bacterial cells, we expressed the TNT from an arabinose-inducible expression vector and IFT from an IPTG-inducible expression vector. Both plasmids were used to co-transform *E. coli* BL21 cells and the transformation was confirmed by PCR (Supplementary Fig. 2a). Schematic representation depicts the experimental procedure performed to study the effect of NAD^+^ modulation on bacterial propagation (Supplementary Fig. 2b). The grown co-transformed cells were plated on agar plates containing IPTG or arabinose or both. We observed that TNT expressing cells on the arabinose plate were able to form less colonies as compared to uninduced control. Interestingly, colony formation was restored on agar plates containing both IPTG and arabinose (Fig. 1b). To evaluate the effect of TNT expression on bacterial growth, the optical density of the cell cultures was measured 1 h after induction with arabinose, IPTG, and both. We found cessation of growth in culture expressing TNT alone. In contrast, culture expressing both TNT and IFT was found to have a similar growth rate as IFT alone expressing culture (Fig. 1c). These results were further confirmed by detecting a significant decrease in the NAD^+^ pool in bacterial cells where TNT alone was induced, compared to the control and IPTG co-induced cells, that displayed significant rescue through co-induction of IFT (Fig. 1d). This clearly indicates that elevation in intracellular NAD^+^ levels resulting from IFT-induction protects bacterial cells from the toxic effect of TNT.

### Determination of NAD^+^-glycohydrolase activity of purified TNT and its inhibition through IFT

Recombinant proteins IFT (rIFT) and TNT (rTNT) were expressed and purified individually (Supplementary Fig. 2c). To purify large amounts of rTNT, the protein was co-expressed with rIFT. rTNT was then separated by heat denaturation of rIFT from the purified IFT-TNT complex (Supplementary Fig. 2d–f). Antibodies were raised against purified rIFT and rTNT proteins (Supplementary methods). We next assessed the binding of rTNT to the rIFT protein by in vitro interaction studies using enzyme-linked immunosorbent assay (ELISA) and Far-Western analysis. Both of these studies confirmed strong binding of IFT with TNT protein (Fig. 1e, Supplementary Fig. 3a). We further demonstrate that rTNT purified from IFT-TNT complex, but not from inclusion bodies, could hydrolyse NAD^+^ (Supplementary Fig. 3b) while rIFT inhibits the NAD^+^ hydrolysis activity of rTNT (Fig. 1f) confirming the antitoxin activity of IFT.

### Intracellular NAD^+^ levels regulates macrophage cell survival

**Fig. 2:**
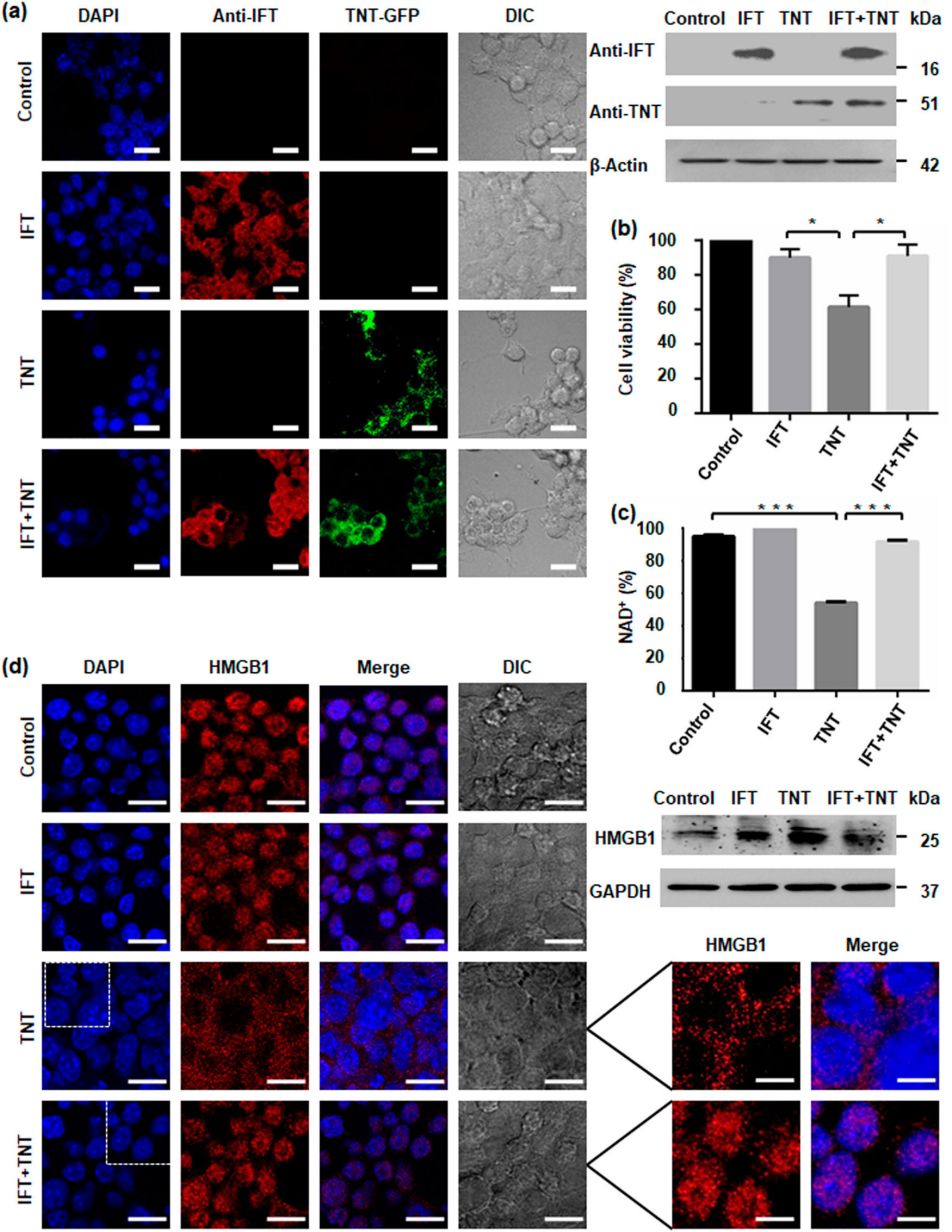
Intracellular NAD^+^ levels regulate macrophage cell survival. **a** Confocal microscopy images of RAW 264.7 cells expressing GFP-TNT or FLAG-IFT or both proteins after 48 h of post-transfection. Nucleus was visualized using DAPI (4,6-diamidino-2-phenylindole). TNT expression was detected by GFP fluorescence and for IFT, cells were immunostained using anti-IFT antibody. Western blot analysis of transfected cells was performed with respective IFT or TNT antibodies. β-Actin was used as a loading control. Scale bar 25 μm, enlarged 10 μm. **b** MTT assay to determine cytotoxicity of TNT expression on transfected cells after 48 h of post-transfection. Cytotoxicity is measured as loss of viability. Increase in cell viability confirms that IFT inhibits TNT-mediated cell death. Cell viability of vector transfected sample was considered as 100%. Statistical significance was quantified using the unpaired t-test with Welch’s correction, *p < 0.05. **c** Intracellular NAD^+^ content of transfected RAW 264.7 cells after 48 h of transfection; the relative percent NAD^+^ level was determined by enzyme-coupling assay. **d** Intracellular translocation of HMGB1 through confocal microscopy. Transfected murine macrophage RAW 264.7 cells were stained with anti-HMGB1 antibody and DAPI. Inhibition of TNT-mediated cell death by IFT expression reduces cytosolic translocation of HMGB1 in co-transfected macrophages. Western blot analysis of HMGB1 was performed in culture supernatant of transfected cells. GAPDH is used as loading control. TNT expression induces HMGB1 translocation and extracellular release which is reverted by co-expression of IFT. Enlarged image showing HMGBI translocation. Scale bar 25 μm, enlarged 10 μm.

To study whether the IFT mediated recovery of endogenous NAD^+^ levels could rescue macrophage cell survival, we transiently transfected RAW 264.7 mouse macrophages with the TNT-EGFP fusion plasmid or IFT-pCMV4_nn_ plasmid or both and monitored expression of TNT and IFT by immunofluorescence assay after 48 h of transfection. We found expression of TNT and IFT in the respective transfected macrophages (Fig. 2a, left). This result was further validated by western blotting and semi quantitative RT-PCR (Fig. 2a, right, Supplementary Fig. 4a). To investigate the effect of NAD^+^ homeostasis on macrophage cell survival, cell viability was determined in transfected macrophages after 48 h of transfection using the MTT assay. We found that TNT expression could significantly decrease cell viability while simultaneous expression of IFT with TNT significantly increased cell viability similar to control macrophages (Fig. 2b). For further confirmation, cell death was analysed in transfected macrophages through uptake of propidium iodide after 48 h of transfection and we observed that TNT expression increased the number of propidium iodide positive cells that was reverted by co-expression of IFT in macrophage cells (Supplementary Fig. 4b). We further determined NAD^+^ levels in transfected macrophages where TNT expression significantly reduces intracellular NAD^+^ pool while IFT co-expression recovers NAD^+^ levels (Fig. 2c). Next, we investigated the localization of HMGB1, as a marker of programmed necrosis^27^, in transfected macrophages by confocal microscopy after 48 h post-transfection and found that while it was co-localized with the nuclei in IFT-transfected macrophages, it was distributed throughout the cytoplasm in TNT transfected macrophages (Fig. 2d, left). Interestingly, in the IFT-TNT co-transfected macrophages, HMGB1 was found to be localized only in nucleus (Fig. 2d lower panel). The above results were further confirmed by western blotting of cytoplasmic and nuclear extracts of different sets of transfected macrophages wherein we observed the same pattern for the localization of HMGB1 (Supplementary Fig. 4c). In addition, HMGB1 levels in the culture supernatant of co-transfected macrophages were also found to be reduced in comparison to TNT-transfected macrophages (Fig. 2d, right). TNT-expressing macrophage cells shows similar pattern of HMGB1 release as described for *Streptococcus pyogenes* NAD^+^-glycohydrolase^28^. These data indicate that restoration of intracellular NAD^+^ levels by IFT inhibits TNT induced macrophage cell death.

### Host intracellular NAD^+^ levels mediates *M. tuberculosis* pathogenesis

**Fig. 3:**
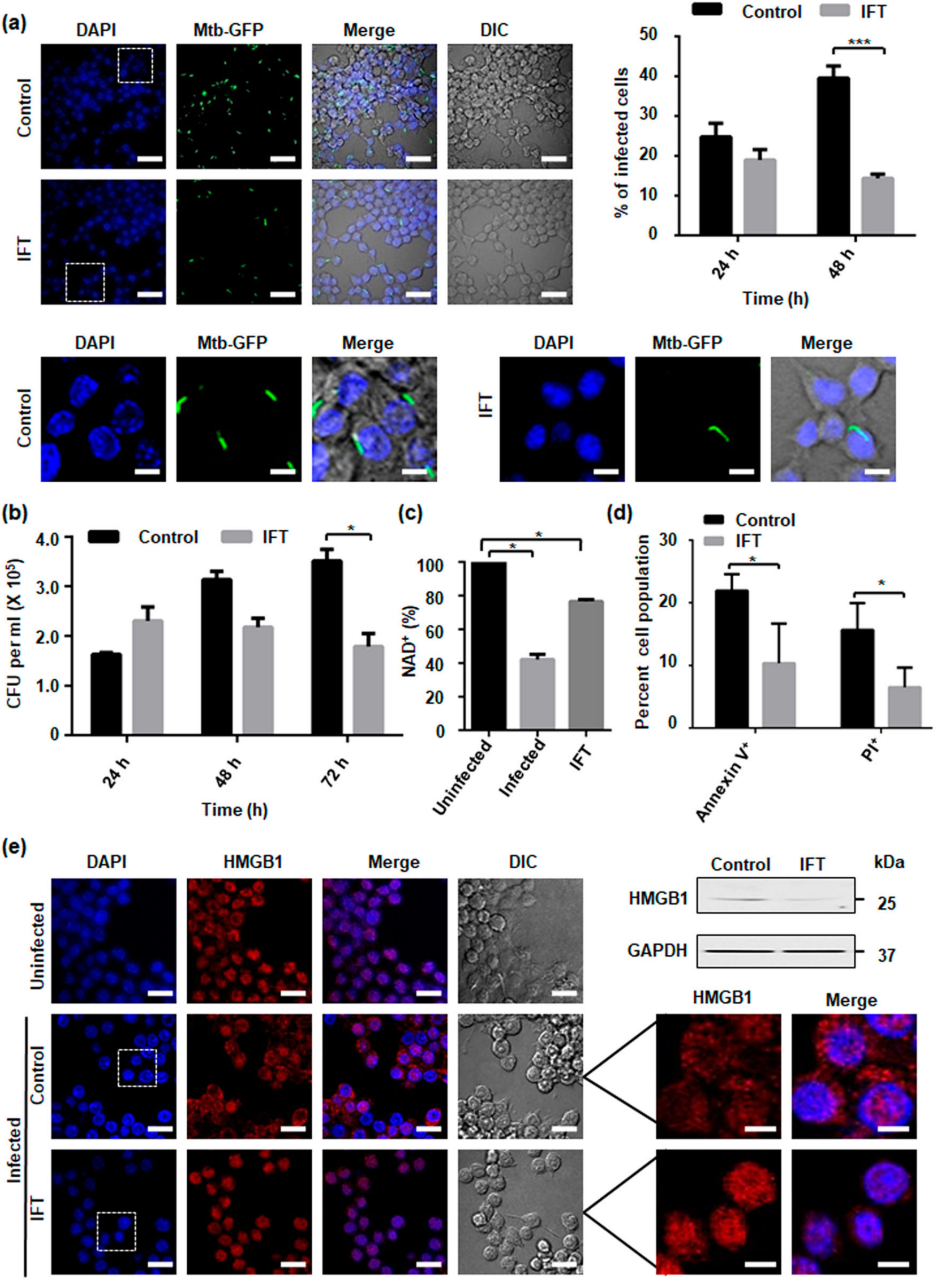
Host intracellular NAD^+^ levels mediate *M. tuberculosis* pathogenesis. RAW 264.7 cells IFT transfected and control were infected with *M. tuberculosis* H37Rv or H37Rv-GFP strain at MOI of 10:1 and analysed 48 h after infection or at the indicated time points. **a** Representative image of confocal microscopic analysis of H37Rv-GFP-infected RAW 264.7 cells. Nuclei were stained with DAPI. Percentages of H37Rv-GFP-infected RAW 264.7 cells expressing IFT were compared to infected control. Significance of difference in percent infection of IFT infected cells with control was calculated using unpaired *t*-tests with Welch’s correction. ***P < 0.001. Scale bar 50 μm, enlarged image 10 μm. **b** Viable intracellular bacterial count of *M. tuberculosis* (colony forming units [CFUs] per millilitre). Decrease in *M. tuberculosis* intracellular growth from 24 h to 72 h post-infection. *p < 0.05, calculated using unpaired *t*-tests with Welch’s correction compared with vector alone transfected infected control at 72 h post-infection. Data are representative of three independent experiments. **c** Intracellular NAD^+^ content of *M. tuberculosis* H37Rv-infected RAW 264.7 cells at an MOI of 10:1 after 48 h of post-infection, Relative percent NAD^+^ level was determined by enzyme-coupling assay in IFT transfected infected cells and control infected cells, in comparison to uninfected cells where NAD^+^ level of uninfected cell was considered as 100%. The significance of inhibition of NADase activity of *M. tuberculosis* was calculated by unpaired t-tests with Welch’s correction, *p < 0.05. **d** Macrophage cell death analysis after 48 h post-infection through flow cytometry by Annexin-V and PI staining of *M. tuberculosis* infected macrophage cells expressing IFT and compared with *M. tuberculosis* infected control cells transfected with vector only, *p < 0.05. **e** Intracellular nucleo-cytoplasmic translocation of HMGB1 in *M. tuberculosis* infected RAW 264.7 cells transiently transfected with IFT compared to uninfected and *M. tuberculosis* infected cells using confocal microscopy after 48 h of infection. HMGB1 was immunostained using anti-HMGB1 antibody and a secondary anti-rabbit Alexa Fluor 594 antibody. Scale bar 25 µm. Evaluation of HMGB1 level in the culture supernatant by western blotting. IFT expression protects macrophages from *M. tuberculosis* induced cell death. Insets show magnifications of cells, highlighting the nuclear and cytoplasmic distribution of HMGB1. Scale bar represents 10 µm.

To explore the possibility whether restoring intracellular NAD^+^ levels by targeting TNT could affect *M. tuberculosis* survival inside the macrophage, we examined the infection pattern of *M. tuberculosis* H37Rv expressing EGFP in murine macrophages transiently transfected with IFT-pCMV4_nn_ construct through confocal microscopy. We observed that the number of *M. tuberculosis* infected cells significantly decreased overtime in IFT-expressing macrophages (Fig. 3a). We next analysed the intracellular survival of *M. tuberculosis* H37Rv through CFU-count and observed significant reduction in intracellular bacterial load after 72 h of infection in IFT-transfected macrophages compared to the control (Fig. 3b). In order to confirm that the effect on intracellular *M. tuberculosis* survival was dependent on cellular NAD^+^ levels, we measured NAD^+^ levels in IFT-expressing and control macrophages after 48 h of *M. tuberculosis* infection. Interestingly, *M. tuberculosis* infected IFT-expressing macrophages showed significantly higher NAD^+^ levels compared to *M. tuberculosis* infected control macrophages (Fig. 3c). Finally, to observe whether *M. tuberculosis* mediated NAD^+^ depletion is associated with macrophage cell death and can be reverted through IFT expression, we assessed cell death in infected control and IFT-expressing infected macrophages using Annexin-V and PI staining. The *M. tuberculosis* infected macrophages demonstrated increased staining of Annexin-V and PI which could be reverted by expression of IFT. Thus, this data suggests that IFT expression inhibits death of infected macrophages (Fig. 3d). We next examined nuclear to cytoplasmic HMGB1 translocation in macrophages by confocal microscopy and western blot analysis (Fig. 3e). We observed HMGB1 translocation from nucleus to cytoplasm in infected macrophages which is inhibited by IFT expression (Fig. 3e, left). The results showed HMGB1 levels in the culture supernatant of IFT expressing infected macrophages to be significantly decreased compared to infected control (Fig. 3e, right). Levels of HMGB1 were also determined in nuclear and cytosolic fractions and significant reduction of nuclear HMGB1 was observed in infected control, which was recovered through expression of IFT in macrophages (Supplementary Fig. 4d). Schematic representation of effect of NAD^+^ modulation on *M. tuberculosis* survival is shown in Supplementary Fig. 4e. Together, these results exemplify our argument that NAD^+^ restoration leads to increased survival of infected macrophages that further reduces the viability of intracellular *M. tuberculosis*.

### NAD^+^ modulation induces premature eryptosis in human erythrocytes

**Fig. 4:**
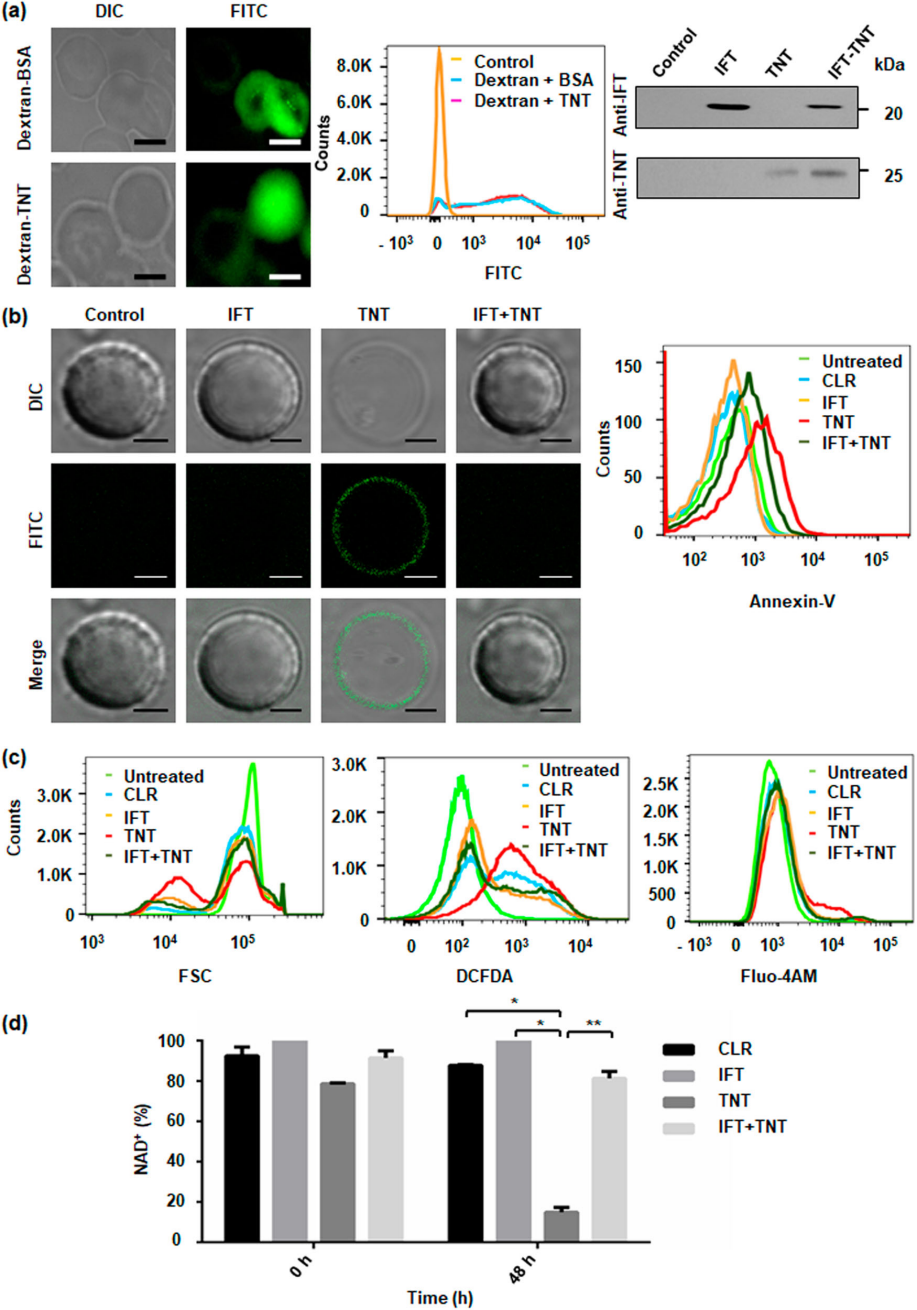
NAD^+^ modulation induces premature eryptosis in human erythrocytes. **a** Imaging of FITC-dextran-loaded erythrocytes with BSA or rTNT proteins at 2 h post-resealing showed efficient loading of cargo. Protein specific confirmation of loaded erythrocytes with respective antibodies after 48 h of incubation at 37 °C; Scale bar 5 μm. **b** Evaluation of eryptosis by confocal microscopy images, of protein-loaded erythrocytes stained with FITC-Annexin-V. Scale bar 4 μm. TNT-loaded erythrocytes exhibit enhanced eryptosis. Representative histograms of AnnexinV-binding of erythrocytes after 48 h of incubation are shown. **c** Histogram of forward scatter representing the decrease in cell volume of rTNT-loaded erythrocytes from control and rIFT-loaded erythrocytes (left). Representative histograms of DCFDA-fluorescence to determine oxidative stress in loaded erythrocytes (middle) are depicted. Representative histograms of Fluo-4AM fluorescence, in rTNT-loaded erythrocytes from control and rIFT-loaded erythrocytes (right) are shown. **d** Intra-erythrocytic NAD^+^ content in loaded erythrocytes before incubation (at time 0) and after 48 h of incubation at 37 °C. Statistical significance of difference was calculated using unpaired t-tests with Welch’s correction, *P < 0.05, **P < 0.01.

We next investigated role of intracellular NAD^+^ modulation in human erythrocytes. To modulate erythrocytic NAD^+^ pool, we used TNT and IFT as molecular tools and loaded erythrocytes with rIFT and rTNT proteins (Supplementary Fig. 5a). To evaluate the efficiency of encapsulated cargo, erythrocytes loaded with rTNT or BSA and 70-kDa FITC-dextran were analysed by confocal microscopy and FACS analysis. The result showed that 99% of all cells were homogenously loaded with the cargo. Furthermore, western blot analysis also confirmed that resealed erythrocyte ghosts stably retain protein cargo (Fig. 4a).

The main characteristics of eryptosis include cell shrinkage, membrane blebbing, exposure of phosphatidylserine on the cell membrane and increase in cytosolic calcium (Ca^2+^) ion levels^23,24^. To determine exposure of phosphatidylserine, FITC-labelled annexin-V binding on the erythrocyte surface of loaded erythrocytes was visualized using confocal microscopy. Annexin-V binding was observed on the surface of rTNT loaded erythrocytes (Fig. 4b left). PS exposure and cell shrinkage was simultaneously quantified using FACS analysis. After 48 h a marked increase in the percentage of annexin-V positive erythrocytes was observed (Fig. 4b right, Supplementary Fig. 5b). Thus, TNT mediated NAD^+^ depletion significantly increases the percentage of phosphatidylserine exposing erythrocytes. This result was further validated by decrease of the forward scatter of rTNT-loaded erythrocytes which reflects erythrocyte shrinkage following 48 h of incubation (Fig. 4c left, Supplementary Fig. 5c).

Further experiments addressed the influence of NAD^+^ depletion on ROS utilizing 2′,7′-dichlorodihydrofluorescein diacetate (DCFDA). Significant increase in DCFDA fluorescence was observed in rTNT-loaded erythrocytes compared to other controls (Fig. 4c middle, Supplementary Fig. 5d).

Next, we investigated whether NAD^+^ depletion could induce cytosolic Ca^2+^ activity. To achieve this, Fluo-4AM fluorescence was taken as a marker of cytosolic Ca^2+^ activity. rTNT-loaded erythrocytes showed a marked increase cytosolic Ca^2+^ activity in comparison to control and rIFT-loaded erythrocytes (Fig. 4c right, Supplementary Fig. 5e).

We then examined the levels of NAD^+^ in loaded erythrocytes. Indeed, loading of erythrocytes with rTNT decreased the NAD^+^ levels by approximately 80 percent after 48 h of incubation (Fig. 4d). Alteration of intracellular NAD^+^ content mirrored the alterations of phosphatidylserine exposure and also buttressed by other hallmarks measured, suggesting that depletion of intracellular NAD^+^ levels induces death of erythrocytes.

### Host erythrocyte NAD^+^ modulation affects parasitic growth

**Fig. 5:**
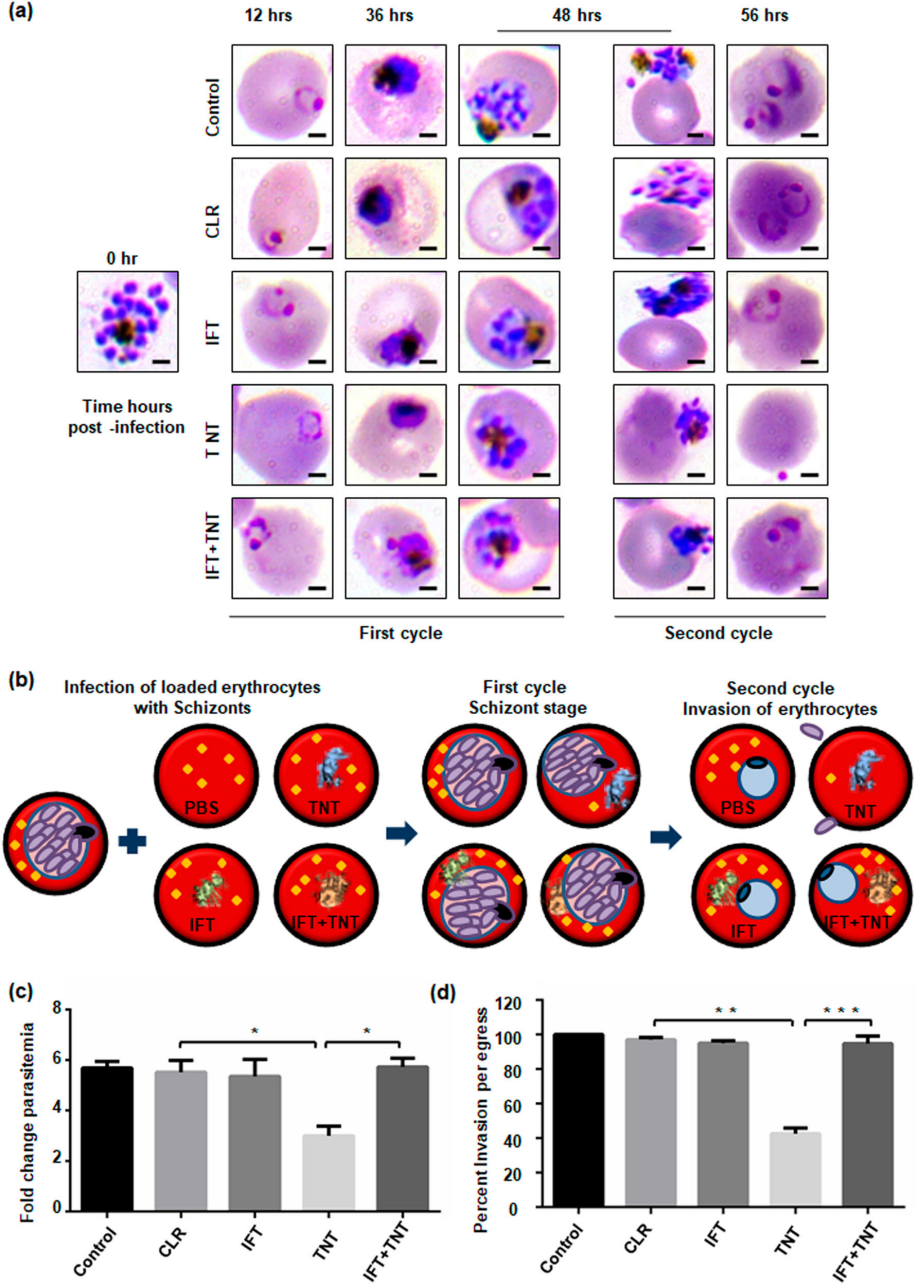
Host erythrocyte NAD^+^ modulation affects parasitic growth. Resealed erythrocytes were infected with mature schizont stage parasites and the parasitemia estimated at different time-points by Giemsa-stained blood smears along with counting of ring- or schizonts-infected erythrocytes. **a** Light microscopy images of Giemsa-stained blood smears at different time points. Scale bar 2 μm. **b** Schematic representation of the proposed mechanism of parasite infection in loaded erythrocytes. **c** Fold-change in parasitemia of each sample after 48 h of infection compared to control at the time of infection. **d** Evaluation of merozoite invasion after 56 h into erythrocytes loaded with respective recombinant proteins. Invasion observed in untreated erythrocytes was considered as 100%. Invasion was reduced by 60% in rTNT-loaded erythrocytes that was successfully recovered with rIFT. The data represent mean of three independent experiments and error bar shows standard deviation. Statistical significance of difference was calculated using unpaired *t*-test with Welch’s correction (*P < 0.05, **P < 0.01, ***P < 0.001).

To evaluate the impact of intra-erythrocytic NAD^+^ levels on *P. falciparum* growth, loaded erythrocytes were incubated with schizonts and monitored for parasite growth at different time points. The control erythrocytes supported parasite growth normally; while the rTNT loaded erythrocytes demonstrate reduced intra-erythrocytic growth of *P. falciparum*. Microscopic analysis of culture smears demonstrated significantly lesser number of new rings in TNT-loaded erythrocytes with increased number of merozoites attached to erythrocytes (Fig. 5a). Schematic representation of the proposed mechanism of parasite infection in loaded erythrocytes is shown in Fig. 5b. Around 2.5 fold decrease in parasitemia was observed in rTNT-loaded erythrocytes as compared to control erythrocytes (Fig. 5c). Percent survival of *P. falciparum* in loaded erythrocytes was also examined by flow cytometry using ethidium bromide staining and this data also demonstrates that parasite survival is significantly reduced in rTNT loaded erythrocytes (Supplementary Fig. 5f). Fold change in parasitemia and Giemsa analysis suggested merozoites invasion defect to host erythrocytes in second cycle of infection. To confirm this hypothesis, loaded erythrocytes were infected with late schizonts and the number of ring formed per schizont egress was scored after 56 h. We observed drastic decrease in the number of ring formed after 56 h in rTNT-loaded erythrocytes that was significantly reverted in the presence of IFT (Fig. 5d). Therefore, these results support that reduced NAD^+^ levels of host erythrocytes could not support the intra-erythrocytic growth of malaria parasites with a pronounced invasion defect.

### Development of hybrid small molecules as potential inhibitors against NAD^+^-glycohydrolase activity of TNT

**Fig. 6:**
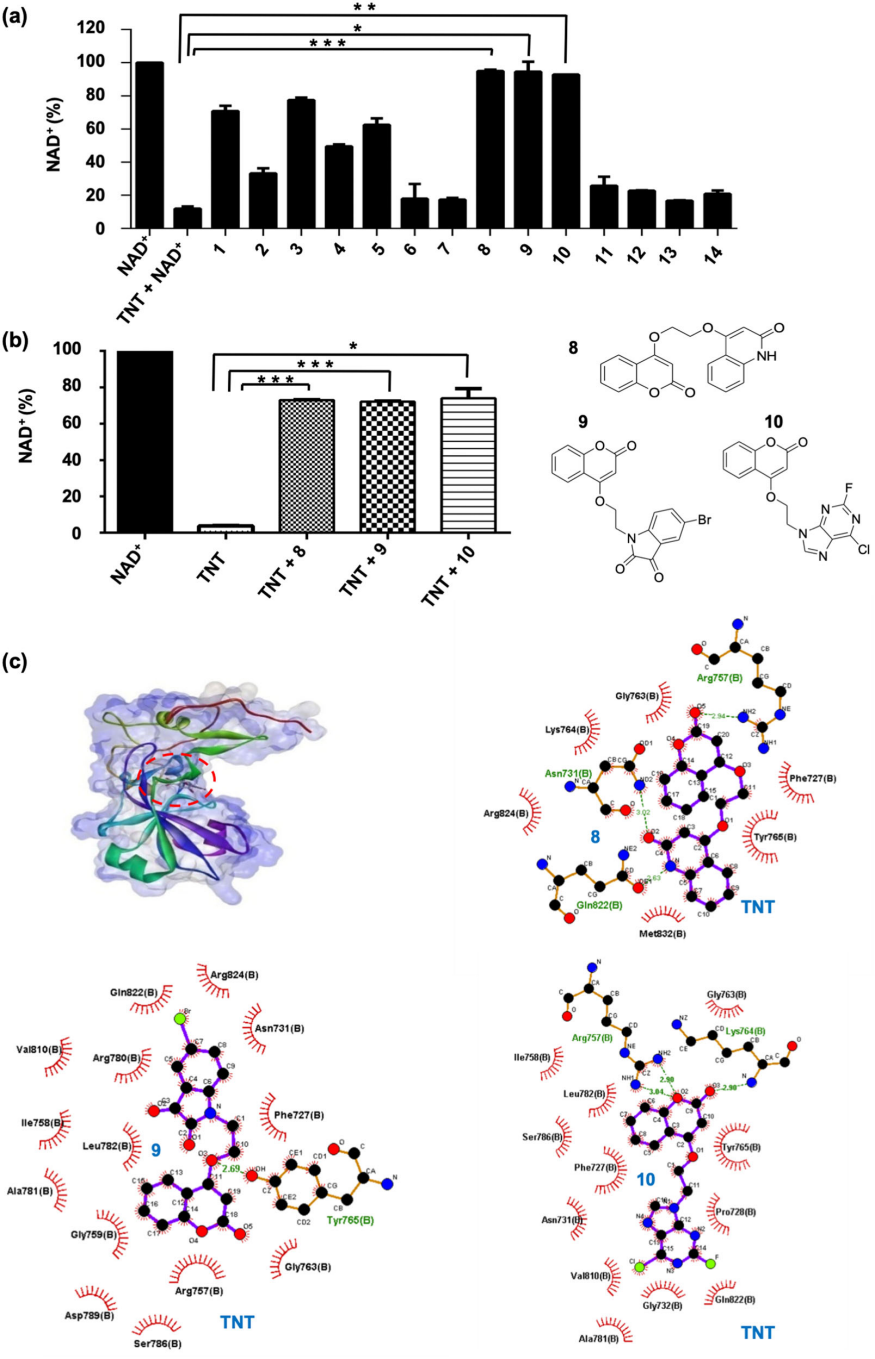
Evaluation of inhibitors against NAD^+^-glycohydrolase activity of TNT. **a** Determination of inhibitory potential of small molecules against NAD^+^-glycohydrolase activity of TNT using NADH fluorescence assay. 10 µM of small-molecule inhibitors was incubated with 75 nM of TNT followed by addition of 100 µM NAD^+^ to initiate the reaction. The remaining NAD^+^ levels were then measured. NAD^+^ level of control reaction without TNT was considered as 100%. Percent decrease in NAD^+^ content represents NAD^+^-glycohydrolase activity. **b** Inhibition of NAD^+^-glycohydrolase activity of TNT by selected molecules **8**, **9**, and **10** using enzyme-coupling assay. Recombinant TNT (30 nM) was incubated with 1 µM of inhibitor followed by addition of 5 µM NAD^+^. NAD^+^-glycohydrolase activity of TNT was measured using enzyme coupling assay. The *P* values were calculated using unpaired *t*-test with Welch’s correction. *P < 0.05, **P < 0.01, ***P < 0.001. Statistical significance was determined considering TNT treated sample as the control against which other groups were compared. **c** 3D surface model of TNT-8, TNT-9, and TNT-10 complex. Ligplot analysis of these interactions and Hydrogen-bonding analysis of TNT-8, TNT-9, and TNT-10 complex showing minimum binding energy of −8.92 kcal/mol, −8.43 kcal/mol, and −7.33 kcal/mol, respectively.

Development of pharmacological molecules against TNT could provide a tool to cure tuberculosis. We designed and synthesized NAD^+^ analogues that could bind to the NAD^+^ binding sites (Supplementary Fig. 6a, Supplementary Table 2). These synthetic molecules were tested for their inhibitory potential against the NAD^+^-glycohydrolase activity of TNT, monitored through the NADH fluorescence assay. The inhibitory effect on the NADase activity was calculated from the decrease of the NAD^+^ amount. Among the molecules tested, **1**, **3**, **4**, and **5** exhibit moderate while **2**, **6**, **7**, **11**, **12**, **13**, and **14** exhibit weak inhibitory activities. On the other hand, molecules **8**, **9**, and **10** showed remarkably good inhibitory potencies (Fig. 6a, Supplementary Table 2). In order to confirm the above results, the inhibitory activity of molecules **8**, **9**, and **10** was also determined using a fluorimetric and highly sensitive coupled-enzyme assay as described in the ‘Methods’ section. Molecules **8**, **9**, and **10** displayed strong inhibition of the TNT NAD^+^-glycohydrolase activity (Fig. 6b). In silico docking of these molecules with the TNT enzyme (PDB code: 4QLP) demonstrated that molecules **8**, **9**, and **10** can bind strongly to the NAD^+^ binding pocket of TNT. These molecules interact with TNT through polar contacts (H bonds) (Supplementary Table 3). Molecules **8** and **9** showed similar binding affinity, higher than the binding affinity of **10** (Fig. 6c). In addition to the NAD^+^ analogues, we also screened the known drugs pyrazinamide (pyrazine analogue of nicotinamide), ribavirin (nucleoside analogue^29^) and APBA (3-aminophenylboronic acid, known inhibitor of NAD^+^-glycohydrolase of *Streptomyces griseus* origin^30^) to determine their inhibitory potential against TNT. APBA inhibited TNT activity by as much as 40%, while ribavirin and pyrazinamide did not inhibit NAD^+^-glycohydrolase activity of TNT (Supplementary Fig. 6b–e). Next, because TNT hydrolyses NAD^+^ into nicotinamide and ADP-ribose, we investigated whether nicotinamide could inhibit TNT activity. We found only 20% reduction in TNT activity in the presence of nicotinamide (Supplementary Fig. 6c, e). Thus the developed NAD^+^ analogues demonstrate better inhibitory potential against TNT activity as compared to the known drugs.

### Effect of NAD^+^ modulation on pathogenic growth

**Fig. 7:**
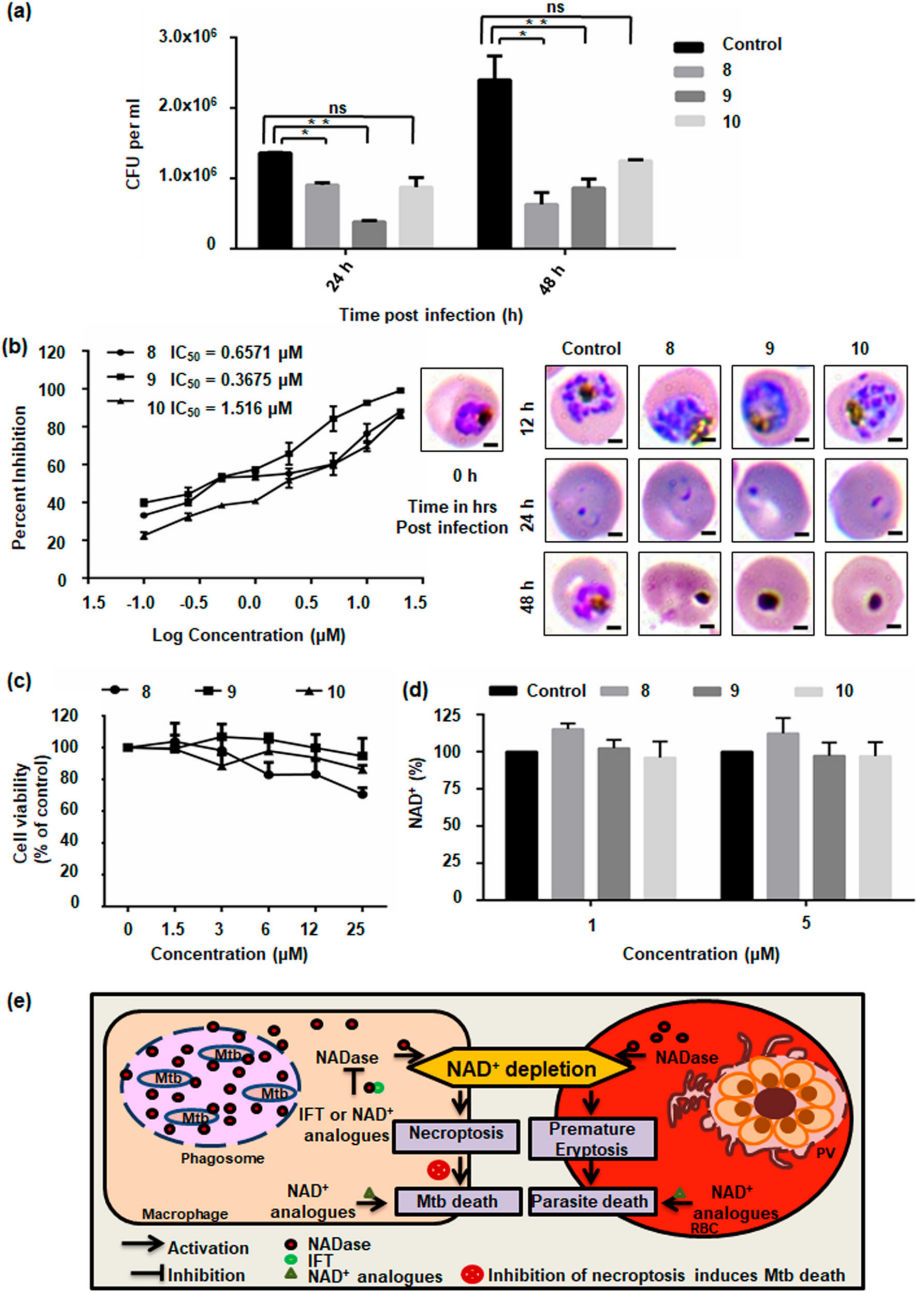
Potential of NAD^+^ modulation on pathogenic growth. **a** Viable intracellular bacterial count of *M. tuberculosis* was determined as CFUs in samples treated with molecules **8**, **9**, and **10**. Significant reduction in CFU was observed in **8** and **9** treated cells at 24 and 48 h post-infection compared to the untreated infected controls (*P < 0.05, **P < 0.01). **b** Growth inhibition assay to calculate IC_50_ value of small molecules **8**, **9**, and **10** from non-linear regression analysis of graph plotted between percent growth inhibition and log concentrations of molecules on graph pad prism 6. Parasite morphology was observed by evaluating Giemsa-stained blood smears under light microscope. Scale bar represents 2 µm. **c** Effect of small molecules **8**, **9**, and **10** on cell viability measured by MTT assay. RAW cells were treated with increasing concentrations (1.5–25 µM) of molecules for 48 h. Results show no significant change in cell viability suggesting that the molecules are not toxic in nature. **d** NAD^+^ levels in erythrocytes treated with 1 µM and 5 µM of small molecules without parasite infection. NAD^+^ level of control erythrocytes was considered as 100%. Significance of Difference in values was calculated using unpaired *t*-test with Welch’s correction (*P < 0.05, **P < 0.01). **e** Model explaining role of NAD^+^ metabolism in pathogenesis of malaria and tuberculosis. Pathogen modulates host NAD^+^ homeostasis for its growth and survival. Targeting this modulation of host NAD^+^ levels could restrict pathogen growth and aid in development of novel drugs.

We next investigated whether these analogues could affect intracellular pathogen survival. *M. tuberculosis* H37Rv infected peritoneal macrophages were treated with these analogues. After 24 and 48 h of treatment, intracellular survival of *M. tuberculosis* was determined by CFU count. Molecules **8** and **9** showed drastic reduction of bacterial load compared to the control. However, only a moderate reduction was noted in the **10-**treated culture (Fig. 7a). Resemblance of these molecules with NAD^+^ suggested that these NAD^+^ analogues could demonstrate anti-malarial activity. We tested the anti-malarial activity of these molecules through growth inhibition assay (GIA) in *P. falciparum* 3D7 strain through counting of Giemsa-stained blood smears. Results indicated **9** to be the most potent molecule with an IC_50_ value of 367.5 nM. Molecules **8** and **10** also exhibited potent anti-malarial activity with IC_50_ values of 657.1 nM and 1.516 μM, respectively. Giemsa staining showed increased number of dead parasites in treated cultures (Fig. 7b). The inhibitory activity of these molecules was also determined through flow cytometric analysis of ethidium bromide stained erythrocytes after 48 h of *P. falciparum* infection, and results showed molecule **9** as most potent inhibitor of *P. falciparum* growth (Supplementary Fig. 7). Furthermore, the cytotoxicity on macrophages of the selected molecules at different concentrations over a 48 h time period was determined using MTT assay. Results showed no difference in cell viability in treated versus untreated cultures, indicating no significant toxicity with respect to the untreated control (Fig. 7c). To rule out the possibility of these molecules affecting NAD^+^ levels of host erythrocytes sans parasite infection, we examined the intra-erythrocytic level of NAD^+^ after treatment with molecules **8**, **9**, and **10**. No significant difference was observed compared to untreated erythrocytes (Fig. 7d). Altogether, these results demonstrate **8** and **9** as potential anti-tubercular and anti-malarial agents. We further conclude that hybrid small molecules represent novel and effective molecules for treatment of malaria as well as tuberculosis and various other NAD^+^ metabolism-mediated diseases.

## Discussion

Intracellular pathogens hijack essential host intracellular pathways to establish infection and spread. Understanding of these pathways can lead to their targeting by small-molecule inhibitors and drugs. One such pathway is NAD^+^ metabolism, and it appears to play a pivotal role in infection propagation^3^. To study the impact of host NAD^+^ modulation on *P. falciparum* and *M. tuberculosis* infection, we used TNT and targeted it by IFT as well as by specifically designed small molecules to explore its druggability.

Stabilizing host NAD^+^ homeostasis is a key factor in determining the fate of intracellular infection^3^. Recently, a seminal study reported role of TNT in host cell death and *M. tuberculosis* infection^6–8^. Our study shows that inhibiting host intracellular NAD^+^ depletion by using TNT’s natural inhibitor IFT restores NAD^+^ levels, prevents cell death, and negatively influences the survival of *M. tuberculosis* inside the macrophage (Fig. 3). These results are supported by a recent study where NAD^+^ replenishment using nicotinamide rescues *M. tuberculosis*-mediated TNT induced host cell death^31^. In addition, we have tested designed novel hybrid small molecules against NAD^+^-glycohydrolase activity of TNT and found that these inhibitors reduce *M. tuberculosis* disease progression. These small molecules are potential drug candidates to treat tuberculosis. Additionally, the effect of NAD^+^ depletion on *E. coli* confirms the universal role of NAD^+^ in determining host cell survival.

Interestingly, intra-erythrocytic progression of *P. falciparum* also depends on induction of eryptosis^25,32^. During the early stage of infection, intra-erythrocytic NAD^+^ levels significantly increase^16^. Here, we show that depleting intra-erythrocytic NAD^+^ levels induces eryptosis. This is consistent with previous reports in the context of other diseases where only NAD^+^ depletion was sufficient for inducing cell death^8^. Although various cell-death patterns are a result of cell-type-specific differences, the cause of eryptosis induction is as yet unclear. Depletion of intra-erythrocytic NAD^+^ can also deprives cellular ATP through impairing glycolysis. ATP depletion has been established previously as a known trigger of eryptosis and inhibit of parasite infection^33,34^. Furthermore, our study could also explain why neoplastic disorders having increased NAD^+^-glycohydrolase activity are associated with a moderate form of anemia in most cases^35^.

The point of contention, whether eryptosis is beneficial or not for parasite progression was also addressed. In the present study, eryptotic early-stage infected rTNT-loaded erythrocytes showed decreased growth of the malaria parasite and these eryptotic erythrocytes are resilient to invasion. These results are in accordance with the previous study where several diseases like sickle-cell anemia, glucose-6-phosphate dehydrogenase (G6PD)-deficiency, beta-thalassemia are strongly associated with accelerated eryptosis and also confer resistance to *P. falciparum* infection^25,36^. Moreover some eryptotic inducers like paclitaxel, chlorpromazine, cyclosporine, curcumin, PGE2, and ionomycin have shown protection against malaria^37,38^. Previous studies have suggested that a reduced expression of host erythrocyte cytoskeleton proteins spectrin and ankyrin, in eryptotic erythrocytes or in ATP depleted erythrocytes, accounts for defect in parasite entry;^34,39,40^ however, our results indicate a role of other host components such as NAD^+^ in the invasion process. Studies have also reported higher NAD^+^ levels in infected erythrocytes compared to uninfected erythrocytes but its exact role remains poorly defined^16,17^. Our findings suggest that adequate NAD^+^ levels are required to be maintained by the parasite in order to restrict the induction of premature eryptosis and facilitate parasite propagation, and that the parasite NAD^+^ pathways can be targeted to develop new therapeutic drugs against malaria without affecting host metabolism.

To explore the potential of NAD^+^ modulation as a drug target in *P. falciparum* and *M. tuberculosis*, we have designed some novel NAD^+^ analogues and showed their inhibitory effect on these pathogens. These analogues do not exhibit cytotoxicity to the host macrophages. Further, these analogues do not affect NAD^+^ levels of host erythrocytes. These confirmations were necessary because the therapeutic significance of NAD^+^ analogues may be limited due to their non-specific cross reactivity with numerous NAD^+^ dependent enzymes present in host cells. NAD^+^ dependent enzymes are well reported in *M. tuberculosis* and studies suggest presence of a large number of NAD^+^-utilizing enzymes^8,41^. However, in comparison to *M. tuberculosis* only two putative NAD^+^-utilizing enzymes, the sirtuin proteins (Sir2), are encoded in *P. falciparum* genome, along with several other NAD^+^-dependent enzymes^42^. That said, the present study does not address the exact mechanisms responsible for the inhibitory effect of the analogues but opens the new avenues to perform elaborate research in this direction.

In conclusion, this study reveals for the first time a novel effect of host NAD^+^ modulation on eryptosis and further establishes its role in the regulation of dissemination of the pathogen from the host cell. Furthermore, reduction in *M. tuberculosis* viability by the TNT natural inhibitor IFT suggests a role of NAD^+^ restoration therapy for the treatment of tuberculosis. More importantly, this study has therapeutic significance in that, by exploiting what appears to be the universal strategy of pathogens, namely NAD^+^ metabolism, our designed analogues show potent anti-tubercular and anti-malarial activity. Further research into these novel NAD^+^ analogues can extend their therapeutic significance for other NAD^+^ mediated diseases.

## Methods

### Reagents

Enzymes were purchased from New England Biolabs or Thermo (Fermentas). Supplementary Table 4 contains the list of *E. coli*, *M. tuberculosis*, *P. falciparum* strains; and plasmids and antibodies used in this study.

### Ethics statement

Animal studies were conducted according to CPCSEA guidelines. Institutional Animal Ethics Committee (IEAC) of JNU has approved the animal studies. BALB/c female mice were maintained under standard conditions and obtained from Central Laboratory Animal Resources, JNU, New Delhi. Donor blood for experiments was obtained from Rotary blood bank (RBB), New Delhi, India.

### Bacterial growth and survival study

The IFT gene (Rv3902c) and the TNT gene (C-terminal domain of CpnT Rv3903c, 648–846 aa) were codon optimized and synthesized by Genscript USA (sequence given in Supplementary Table 1).Genes cloned with the unique *Sna*BI restriction site were obtained in pUC57 vector.

TNT and IFT genes were amplified and then directionally cloned into the *Sna*B1-cut dephosphorylated pMTSA and pET28a vectors. To protect *E. coli* BL21 (DE3) cells from TNT mediated toxicity, the TNT construct was co-transformed with the IFT-carrying plasmid. The clones were screened and confirmed by colony PCR, restriction digestion with *Sna*BI restriction enzyme and direct sequencing using primers that were sequences from the pMTSA and pET28a vectors, respectively.

To test the effect of IFT and TNT expression on the colony formation efficiency of *E. coli* BL21 (DE3) cells, overnight grown cultures of positive co-transformed clones were inoculated (100 fold dilution) and grown till the culture OD_600nm_ reached 0.2–0.3. Different dilutions of the secondary culture were platted on LB agar plates with streptomycin (100 µgml^−1^), kanamycin (50 µg ml^−1^) containing 0.2% arabinose or 25 μM IPTG, or both, to induce expression of TNT, IFT, or TNT along with IFT, respectively. Colony formation was analysed after 16 h incubation at 37 °C.

To study the kinetics of TNT expression on bacterial optical density, secondary cultures of three different colonies of *E. coli* BL21 (DE3) cells expressing both the TNT and IFT genes, were grown in LB medium till an OD_600nm_ of 0.2. Individual cultures were induced with addition of 0.2% arabinose, 1 mM IPTG, or both respectively and incubated at 37 °C to continue to grow. OD_600 nm_ was measured at different time points after induction.

### Expression and purification of rTNT protein

The TNT gene was excised from TNT-pUC57 construct and cloned into dephosphorylated *Sna*BI-cut pMTSA expression vector. Upon induction, TNT-pMTSA construct produces C-terminal hexa-Histidine tagged-TNT protein. To purify the TNT protein, TNT-pMTSA plasmid containing *E. coli* C43 (DE3) cells were grown in glucose supplemented LB medium. At mid-log stage, cells were induced with 0.2% L-arabinose and incubated at 25 °C with constant shaking for 10 h. The induced cells were pelleted and washed with PBS buffer (4.3 mM Na_2_HPO_4_, 1.47 mM KH_2_PO_4_, 150 mM NaCl, 2.7 mM KCl, pH 7.4) and resuspended in buffer for lysing cells (2 mM PMSF in PBS, pH 8.0). After sonication the cell lysate was centrifuged and the pellet fraction solubilised in buffer (PBS buffer with 8 M urea, pH 8.0) by continuous shaking at 25 °C for 12 h. Filtered soluble fraction obtained after centrifugation of the solubilised sample was incubated with Ni-NTA resin for binding (Qiagen, Germany) at 25 °C overnight. Ni-NTA bound protein was eluted using imidazole gradient (8 M urea in PBS buffer with 20 mM to 500 mM imidazole, pH 8.0). The pooled fractions were dialysed against buffer (PBS, pH 8.0, 8-0 M urea) to gradually remove urea. To assess purity, dialysed rTNT protein was run on an SDS-PAGE (Supplementary Fig. 2c). Antibodies were raised against the purified TNT protein (Supplementary methods).

### Expression and purification of IFT

The *Sna*B1-digested IFT insert was cloned at the unique *Sna*B1 restriction site into dephosphorylated *Bla*1cut-pET28a expression vector. Upon induction, IFT-pET28a construct produces C-terminal hexa-Histidine tagged IFT protein. For purification of rIFT protein, *E. coli* BL21 (DE3) cells harbouring IFT-pET28a plasmid were cultured till mid-log phase and then induced for 10 h with the addition of 1 mM IPTG at 25 °C. The induced cells were pelleted and resuspended in lysis buffer (2 mM PMSF in PBS buffer, pH 7.4) for sonication. The sonicated lysate was centrifuged and the resultant supernatant was incubated with Qiagen Ni-NTA agarose beads equilibrated with lysis buffer for binding overnight at 4 °C with constant shaking. Ni-NTA beads bound with rIFT protein were washed with buffer (20 mM imidazole, 25 mM Tris, 0.3 M NaCl, and pH 7.4) and bound protein was eluted through imidazole gradient (50–250 mM imidazole, 25 mM Tris, 0.3 M NaCl, and pH 7.4). Purified fractions were dialysed to remove imidazole, quantified and stored at −20 °C (Supplementary Fig. 2c). rIFT protein was also purified in PBS buffer for the purposes of raising anti-IFT antibodies.

### Expression and purification of the IFT-TNT complex

Secondary culture of *E. coli* C43 (DE3) cells carrying both the TNT-pMTSA and IFT-pET28a plasmids was grown till mid-log phage in LB medium containing kanamycin (50 μg ml^−1^), streptomycin (100 μg ml^−1^) supplemented with glucose, and then induced with 0.2% L-arabinose and 1 mM IPTG and further incubated at 25 °C for 12 h. After washing with Tris-NaCl buffer (20 mM Tris, 0.2 M NaCl, and pH 7.4), the induced culture was pelleted and resuspended in lysis buffer (2 mM PMSF containing Tris-NaCl buffer) for sonication. This lysate was then centrifuged and the IFT–TNT complex was found in the supernatant fraction. Supernatant fraction with IFT–TNT complex was incubated with Qiagen Ni-NTA agarose beads for binding with constant shaking at 4 °C overnight. Resin-bound proteins were washed with buffer (20 mM imidazole in Tris-NaCl buffer) and eluted using imidazole gradient (50–200 mM imidazole in 20 mM Tris, 0.2 M NaCl, and pH 7.4). Purified protein fractions were collected and dialysed against Tris-NaCl buffer and the integrity of the protein analysed on SDS-PAGE. Purified protein complex was stored at −20 °C after quantification with Bradford assay (BioRad, USA) (Supplementary Fig. 2f).

### Purification of TNT from IFT–TNT complex for functional assays

Purified IFT–TNT complex (in 20 mM Tris–HCl, 0.2 M NaCl, and pH 7.4) was heated at 55 °C for 20 min to release free TNT, while IFT remained in the insoluble fraction. SDS page analysis of purified protein was performed to assess the purity of protein (Supplementary Fig. 2f).

### In vitro protein–protein interaction

The interaction between purified recombinant TNT and IFT proteins was evaluated by far-western blotting^43^ and ELISA^43,44^ as described previously.

### Far-western blot analysis

Purified bait protein TNT (2 μg) was separated using 12% SDS-PAGE and for further experiment electro-transferred onto the PVDF membrane. As described in protocol previously, briefly, the transferred bait protein TNT was denatured and renatured on the membrane. After blocking with 5% skimmed milk, the membrane with renatured bait protein was overlaid by prey protein IFT (3 μg ml^−1^) in buffer (2% skimmed milk powder with 20 mM Tris (pH 7.6), 0.5 mM EDTA, 100 mM NaCl, 1 mM DTT, 0.1% Tween-20,10% glycerol) for binding and incubated for 2 h at RT. Prey protein IFT was probed with primary mouse raised anti-IFT antibody (1:5000) followed by secondary antibody (dilution 1:5000, HRP-conjugated anti-mouse, Thermo Scientific, USA). Signal was detected by chemiluminescence using the Luminol reagent (Bio-Rad). Human protein ETHE1 (raised in-house; unpublished work) was used as negative control.

### ELISA

Interaction between recombinant proteins IFT and TNT was analysed by ELISA as described previously^43,44^. Briefly, the purified recombinant bait protein TNT at 200 ng concentration in 100 μl was coated in buffer (100 mM sodium carbonate/bicarbonate-coating buffer, pH 9.6) on a 96-well microplate (Nunc MaxiSorp ELISA plates). After washing, wells were blocked with 3% BSA in 1×PBST (0.1% Tween-20 in 1×PBS) at 37 °C for 1 h, and again washed with 1XPBST. Subsequently, prey protein IFT at varying concentrations (50 ng, 100 ng, 200 ng, 500 ng, 1000 ng) in binding buffer (50 mM HEPES, 250 mM Potassium acetate and 5 mM magnesium acetate, pH 8.0) was added to test wells at 37 °C for 1 h. Subsequent to washing, IFT protein was detected by incubation with anti-IFT antibody (mouse polyclonal, 1:15000) for 1 h at 37 °C followed by the secondary antibody (anti-mouse HRP conjugated, 1:5000, Thermo Scientific, USA) for 1 h. The test wells were washed thoroughly and incubated to develop signal for 30 min at 37 °C after addition of HRP substrate TMB to the respective wells. 1N NH_2_SO_4_ solution was used to stop the reaction and at 450 nm the absorbance reading was taken with an ELISA plate reader (TECAN).

### Determination of NAD^**+**^**-**glycohydrolase (NADase) activity of TNT

The NAD^+^-glycohydrolase (NADase) activity of TNT was determined using three different methods. All reagents were from Sigma-Aldrich.

Enzyme assays were conducted by detection of NADH fluorescence at excitation 360 nm and emission 460 nm as reported previously with minor modifications^7^. The inhibitory potential of the small-molecule analogues against NADase activity of TNT was determined by mixing 10 μM of the analogues with recombinant TNT (75 nM) at RT in reaction buffer (0.2 M NaCl, 20 mM Tris, and pH 7.4). 100 μM β-NAD^+^ was added to the mixture and the reaction stopped using 5 M NaOH. Subsequently, after an incubation of 1 h at 37 °C, the relative fluorescence was determined at excitation 360 nm and emission 460 nm on a Thermo Scientific Varioscan multimode microplate reader and compared to a standard NAD^+^ concentration curve. Relative NAD^+^ content was measured with comparison to the control without the analogue inhibitor. Next, to further confirm the results, a fluorimetric and highly sensitive coupled enzyme assay was conducted as previously described, with minor modifications^45^. Briefly, TNT (30 nM) was incubated with small molecules (1 µM) in the dark in the reaction buffer (0.2 M NaCl, 20 mM Tris, and pH 7.4). To start the reaction 5 µM β-NAD^+^ was added and after incubation 150 mM HCl was used to stop the reaction followed by neutralization with sodium phosphate buffer (pH 8). The amount of remaining NAD^+^ was determined using an enzyme cycling reaction^45^ and the rate of increase in fluorescence was measured at excitation 544 nm and emission 590 nm on a microplate fluorometer (Fluoroskan Ascent FL, Thermo Scientific). The slope of fluorescent increase was measured in comparison to the untreated control to determine the relative NAD^+^ content. For determination of NAD^+^ levels in cells, samples were treated with HCl to extract NAD^+^ and after neutralization fluorescence based enzyme coupling assay was performed.

The reaction buffer (0.2 M NaCl, 20 mM Tris-HCl, and pH 7.4) containing β-NAD^+^ and recombinant TNT (30 nM) was incubated at 37 °C. The remaining NAD^+^ content in each sample was measured following the protocol of Bioassay Systems EnzyFluo NAD/NADH Assay Kit.

A fresh aliquot of TNT was used for every assay and all reagents were prepared fresh or stored in aliquots to avoid the freeze-thaw cycle. As NAD^+^ is light sensitive, all incubations with NAD^+^ were conducted in dark and quantification of the protein concentrations was done by Bradford method (Bio-Rad, USA).

### Culture conditions and transfection

For the purposes of expressing the IFT protein in macrophages, the *Sna*B1-digested insert of IFT was cloned into the *Eco*RV-cut and dephosphorylated mammalian expression vector pFLAGCMV4_nn_. Separately, the *Sna*B1-digested insert of TNT, with a single nucleotide addition to allow for it to be in-frame with the EGFP coding sequences, was cloned into *Sma*1-cut and dephosphorylated pEGFPC1 vector.

Murine RAW 264.7 macrophage cell line (ATCC) was maintained in cell culture medium DMEM (Gibco, Life Technologies) containing 2 mM L-glutamine, 10 mM HEPES, 100 IUml^−1^ penicillin, and 100 μg ml^−1^ streptomycin supplemented with 10% FBS with 5% CO_2_ at 37 °C. For transfections, RAW macrophages were seeded into the plate and next day, at ~80% confluency, the macrophages were transiently transfected according to the manufacturer’s protocol using Lipofectamine™ 2000 reagent (Invitrogen, USA). After 48 h post-transfection, the RAW macrophage cells were observed under a fluorescence microscope to measure transfection efficiency and the transfected macrophages used for subsequent experiments. For double transfections, an equal amount of both plasmids were taken, while single plasmids were normalized with respective controls. The plasmids pCMV4_nn_, pEGFPC1 (as control), and TNT-pEGFPC1 and IFT-pCMV4_nn_ as constructs, were used for transient transfections of macrophages. To analyse protein expression, macrophages were resuspended in RIPA buffer for lysis (G-Biosciences, USA) with a protease inhibitor cocktail (Roche diagnostics). After centrifugation at 13,000 rpm for 20 min at 4 °C, soluble fractions were loaded, as quantified using the Bradford method (Bio-rad, USA), to analyse by western blotting using respective antibodies.

### Immunofluorescence and confocal imaging

RAW macrophage cells were cultured on coverslips and after 48 h post-treatment washed with PBS. Subsequently, cells were incubated in PBS solution containing 4% paraformaldehyde for fixation at RT for 15 min. Following washing with cold PBS fixed cells were permeabilized by addition of PBS solution containing 0.2% Triton X-100 for 10 min at RT and subsequently blocked with 1% BSA in PBS solution. After blocking, respective primary antibodies (PBS with 1% BSA) were added to cells at RT for 1 h, followed by incubation with respective secondary antibodies. The stained cells after washing were mounted using DAPI antifade solution (Sigma-Aldrich) and analysed through a confocal microscope (Olympus Corporation, Japan). Image analysis was performed using NIS Elements software.

For the observation of eryptotic erythrocytes, loaded erythrocytes were washed and stained with FITC-annexin-V (Thermo Scientific) at 1:20 dilution in Annexin-V binding Buffer (10 mM HEPES, 140 mM NaCl, 2.5 mM CaCl_2_, and pH 7.4) for 20 min. After washing, mounted erythrocytes were analysed using confocal microscope (Nikon, Japan). 10 kDa FITC-dextran loaded erythrocytes were evaluated for loading efficiency by fluorescence microscope.

### Flow cytometry analysis of cell death

Macrophage cells transfected with TNT-pEGFPC1 and IFT-pCMV4_nn_ constructs were stained with PI (Biolegend, USA) after 48 h of post-transfection. *M. tuberculosis* infected control or IFT expressing infected macrophage cells were stained with FITC-Annexin-V and PI (FITC Annexin-V Apoptosis detection kit with PI, Biolegend, USA) following manufacturers protocol, after 48 h of post-infection with *M. tuberculosis* H37Rv. The stained macrophage cells were analysed by flow cytometry (BD, LSR Fortessa, Becton Dickinson, USA) using FlowJo software (Tree Star Inc, USA).

### Cell viability assay

Transfected RAW macrophage cells with TNT-pEGFPC1 and IFT-pCMV4_nn_ constructs were analysed after 48 h of transfections to determine TNT-induced macrophage cell death. To assess effect of small molecules on cell viability, RAW macrophage cells were treated for 48 h with different concentrations of small molecules **8**, **9** and **10** (1.5 µM, 3 µM, 6 µM, 12 μM, 25 µM). MTT assay was conducted to determine cell viability using MTT reagent [3-(4,5-dimethylthiazol-2-yl)-2,5-diphenyltetrazolium bromide, Sigma-Aldrich]. Viable cells reduce MTT into dark blue farmazon crystals. Cells were incubated with MTT solution (0.5 mg ml^-1^ in PBS) for 3 h at 37 °C. Media was removed after incubation and farmazon crystals were dissolved by addition of 100 µl of DMSO to each well. Absorbance reading at 570 nm was taken using Varioscan multimode microplate reader (Thermo scientific). Percent cell viability was calculated by relative reduction in absorbance values of treated samples compared to the control.

### *M. tuberculosis* H37Rv infection of macrophages and post-infection CFU count

RAW macrophage cells after 24 h of transfections with IFT-pCMV4_nn_ were infected at 10 multiplicity of infection (MOI) with *M. tuberculosis* H37Rv or H37Rv-GFP and incubated for 4 h at 37 °C. The *M. tuberculosis* infected macrophages were washed with DMEM (Gibco,USA) and again incubated in complete medium till respective time points for further experiments. To determine the CFU count, *M. tuberculosis* infected cells were collected after 24, 48 and 72 h of infection and lysed with 0.05% SDS solution in 7H9 medium (Sigma-Aldrich). For colony counting, 7H11 agar plates were plated with the lysate at different dilutions. Plates were analysed and counted after 15 days of incubation. For confocal studies, *M. tuberculosis* H37Rv-GFP infected cells were washed after 48 h of infection and fixed (4% paraformaldehyde) and then subjected to analysis under confocal microscope (Olympus Corporation, Japan). The number macrophages infected with *M. tuberculosis* were counted manually and the percentage of infected macrophages was determined as follows:

*M. tuberculosis* infected macrophages (%) = (Number of infected macrophages/Total number of macrophages) * 100.

### Effect of small-molecules on the intracellular survival of *M. tuberculosis*

Female BALB/c mice (6–7 weeks-old) were intraperitonealy injected with 2 ml of thioglycollate. On the fifth day of infection all mice were sacrificed and macrophages were collected by *peritoneal* lavage. To determine potency of small-molecules against *M. tuberculosis*, isolated intraperitoneal macrophages were seeded and allowed to adhere for 24 h. Following adherence, cells were infected with *M. tuberculosis* H37Rv at 10 MOI for 4 h, and after washing, again incubated with complete medium containing 25 µM of the small-molecules. Cells were harvested at time points 24, 48 h and processed for CFU count.

### Loading of proteins in erythrocytes

For loading of erythrocytes with recombinant proteins a previously described method^46^ was used, briefly, after washing with PBS erythrocytes were resuspended in glucose containing PBS solution at 50 percent hematocrit (50 µl packed erythrocyte used per sample). After centrifugation, packed erythrocytes were chilled on ice for 5 min and an equal volume of lysis buffer (1 mM ATP, 5 mM K_2_HPO_4_, final pH 7.4) was added for lysis, followed with incubation on ice for 1 h. Protein cargos (5 µg rIFT, 5 µg rTNT, or rIFT with rTNT 5 µg each in PBS per 50 µl packed erythrocytes) were added to the lysis buffer. Control sample contained only PBS. Lysed erythrocytes were subsequently resealed by including 25 µl of concentrated resealing buffer (1 mM ATP, 237.5 mM KCl, 475 mM KOAc, 25 mM MgCl_2_, and 25 mM Na_2_HPO_4_, final pH 7.0) to lysing erythrocytes and for 1 h incubated at 37 °C. Resealed erythrocytes samples were three times thoroughly washed with incomplete RPMI medium. Untreated and control samples were included for comparison. All reagents were from Sigma-Aldrich. FITC-Dextran 10,000 MW (Sigma-Aldrich) with protein (BSA or rTNT) was used to determine loading efficiency.

### Flow cytometric assays to determine eryptosis

Flow cytometer (BD, LSR Fortessa, Becton Dickinson, USA) was used to perform flow cytometric studies and data was analysed through FlowJo software (Tree Star Inc, USA).

### Evaluation of FITC-dextran loaded erythrocytes

To determine loading efficiency, erythrocytes were loaded with 10 kDa FITC dextran and after washing resuspended in PBS-Glucose solution. Fluorescence intensity of FITC was measured using flow cytometry (BD, LSR Fortessa).

### Determination of PS exposure and forward scatter

To measure eryptosis in loaded erythrocytes, erythrocytes were incubated with FITC-annexin-V (dilution 1:20, Thermo Scientific) in Annexin-V binding Buffer (10 mM HEPES, 2.5 mM CaCl_2_, 140 mM NaCl, and pH 7.4) at room temperature for 20 min. After washing, erythrocytes were analysed by flow cytometry (BD, LSR Fortessa) at 488/530 nm (excitation/emission wavelength). Loaded erythrocytes were also measured for forward scatter through flow cytometry.

### Quantification of intracellular calcium level

For the determination of intracellular Ca^2+^ level, Fluo-4AM (Life Technologies, USA) was added to the resealed erythrocytes. Loaded erythrocytes were analysed for intracellular Ca^2+^ level by measuring fluorescence intensity with flow cytometry (BD LSR Fortessa) at 488/530 nm (excitation/emission wavelength).

### Reactive oxygen species (ROS) measurement

For quantification of reactive oxygen species (ROS), erythrocytes were incubated with 2,7-dichlorofluorescein diacetate (DCFDA, Sigma-Aldrich) for 25 min at 37 °C. Subsequent to washing, the stained erythrocytes were measured for ROS-dependent fluorescence intensity using flow cytometry (BD LSR Fortessa) at 488/530 nm (excitation/emission wavelength).

### Parasite culture

In vitro *P. falciparum* 3D7 strain was maintained using O^+^ erythrocytes in RPMI culture medium (Gibco,USA) supplemented with 2 mM l-glutamine containing 0.5% Albumax II, 27.2 mg L^−1^ hypoxanthine, 25 mM HEPES, 25 mM sodium bicarbonate and, final pH 7.4, in an incubator at 37 °C and by maintaining a mix gas environment (90% N_2_, 5% CO_2_, and 5% O_2_). Synchronization of parasite culture was achieved through sorbitol treatment. Schizonts were purified by percoll treatment. Routine parasitemia and staging were observed by Giemsa staining (Sigma-Aldrich).

### In vitro *P. falciparum* growth inhibition assay

To evaluate the inhibitory potential of the small molecules on *P. falciparum* growth, early trophozoite stage synchronized culture of the parasite at a parasitemia of 1% and 2% hematocrit was aliquoted in 96-well plates (Nunc,Thermo, USA) and treated with different concentrations of the molecules **8, 9**, and **10** (100 nM–20 µM) for 48 h. Following incubation, Giemsa-stained blood smears were observed to assess the parasitemia. Controls include untreated and DMSO-treated cultures. IC_50_ was determined by graph pad prism 6.0 (CA, USA). Percent inhibition of *P. falciparum* growth was determined as follows:

Percent Inhibition = (1 − Percent parasitemia of treatment/ Percent parasitemia of Control) ∗ 100.

### *P. falciparum* invasion assay

Percoll-purified late-stage schizonts were used to infect protein-loaded erythrocytes while maintaining 1% parasitemia and 2% hematocrit. Giemsa-stained thin-blood smears were prepared at different time-points and evaluated for total parasitemia as well as number of rings and schizonts present. To determine parasite invasion, number of rings produced from single schizont was counted. Percent invasion was defined in treated samples as a percentage of number of rings produced per schizont relative to untreated control^47,48^. Invasion in untreated control was considered as 100%. Fold change in parasitemia was determined after 48 h of infection by dividing final parasitemia with initial parasitemia of each sample.

### In-silico docking studies

3D protein structure of *M. tuberculosis* protein TNT was obtained (PDB ID-4QLP, IFT-TNT complex structure) from Protein databank. Swiss PDB viewer and chem.Bio Draw ultra 3D software were used to optimize protein and ligands structure. Molecular docking of molecules 8, 9, and 10 with TNT was performed using Autodock version 4.2 and Cygwin terminal version 3.1 software. Residues involved in NAD^+^ Binding and hydrolysis were chosen to prepare grid around to perform ligand binding. UCSF Chimera version 1.14, Ligplot^+^ version 2.2, Discovery Studio version 19.1.0 and Pymol version 2.3.2 software were utilized for further analysis and visualization of docking results as described previously^49^.

### Statistical analysis

The results represent mean values of three independent experiments and error bar represented standard deviation of data. Unpaired two-tailed Student’s *t* test was conducted to measure *P* values. Calculated *P* values which were less than 0.05 reported as statistically significant, where *p* values < 0.05 denotes *, < 0.01 denotes **, and < 0.001 denotes ***.

### General experimental methods for small hybrid molecules

All synthetic experiments were performed in an oven-dried apparatus. High resolution mass spectra obtained from a quadrupole/TOF mass spectrometer with an ESI source. Solvents were distilled by standard distillation procedure and stored in 4 Å and 3 Å molecular sieves. ^1^H (400 MHz), ^13^C (100 MHz) NMR spectra was recorded with a Bruker AMX-400 MHz instrument. ^1^H and ^13^C chemical shifts are referenced to the solvents residual signals D_2_O ^1^H NMR δ 4.79, DMSO ^1^H 2.50 and δ 39.52 for ^13^C and CD_3_OD, ^1^H NMR δ 4.87, 3.31 and ^13^C δ 49.00 reported in parts per million (ppm) at 25 °C. Coupling constants are expressed in hertz (Hz). Reactions were monitored by thin-layer chromatography carried out on 0.25 mm E. Merck silica gel plates (60F-254), spots were visualized by phosphomolybdic acid and 10% H_2_SO_4_ in ethanol. All the reagents used in the preparation of small hybrid molecules were purchased from Sigma-Aldrich and used under good laboratory practice.

## Supplementary information

Supplementary figure legends

Supplementary table 1

Supplementary table 2

Supplementary table 3

Supplementary table 4

Supplementary table 5

Supplementary Figure 1

Supplementary Figure 2

Supplementary Figure 3

Supplementary Figure 4

Supplementary Figure 5

Supplementary Figure 6

Supplementary Figure 7
