# Supplementary material for: Pathogen induced subversion of NAD+ metabolism mediating host cell death: a target for development of chemotherapeutics
Source: Cell Death Discov. 2021 Jan 13;7:10. doi: 10.1038/s41420-020-00366-z (PMC7806871; doi:10.1038/s41420-020-00366-z)
Supplement: Supplementary file 1 — Supplementary figure legends [file 41420_2020_366_MOESM1_ESM.docx]

**Supplementary Information**

**Pathogen induced subversion of NAD^+^ metabolism** **mediating host cell death: a target for development of chemotherapeutics**

**Ayushi Chaurasiya*#^1^, Swati Garg#^1^, Ashish Khanna#^2^, Chintam Narayana^2^, Vedprakash Dwivedi^3^, Nishant Joshi^4^, Zill e Anam^1^, Niharika Singh^1^, Jhalak Singhal^1^, Shikha Kaushik^1^, Amandeep Kaur^1^, Pallavi Srivastava^1^, Manisha Marothia^1^, Mukesh Kumar^1^, Santosh Kumar^3^, Geeta Kumari^1^, Akshay Munjal^1^, Sonal Gupta^1^, Preeti Singh^1^, Soumya Pati^4^, Gobardhan Das^1^, Ram Sagar^2^*, Anand Ranganathan^1^* and Shailja Singh^1^***

*^1^Special Centre for Molecular Medicine, Jawaharlal Nehru University, New Delhi-110067,India*

*^2^Department of Chemistry, Institute of Science, Banaras Hindu University, Varanasi 221005, Uttar Pradesh, India*

*^3^International Centre for Genetic Engineering and Biotechnology, New Delhi-110067, India*

*^4^Department of Life Sciences, School of Natural Sciences, Shiv Nadar University, Greater Noida-201314, India*

^*^*Email:* [*shailja.jnu@gmail.com*](mailto:shailja.jnu@gmail.com)*,* [*ram.sagar@bhu.ac.in*](mailto:ram.sagar@bhu.ac.in)*,* [*anand.icgeb@gmail.com*](mailto:anand.icgeb@gmail.com)*,* [*ayushi.chaurasiya01@gmail.com*](mailto:ayushi.chaurasiya01@gmail.com)*.*

# These authors contributed equally to this work as joint first authors.

**Supplementary Fig 1.** Toxin-Antitoxin complex of *M. tuberculosis* (TNT-IFT)*.* Complete amino acid sequence of IFT and channel protein with necrosis-inducing toxin (Cpnt). Highlighted region represents tuberculosis necrotizing toxin (TNT, protein amino acid sequence used for present study). Protein parameters highlighting length, molecular weight (MW), isoelectric point (pI) and grand average of hydropathicity (GRAVY) of interacting proteins IFT and TNT.

**Supplementary Fig 2.** Co-expression of IFT with TNT rescues bacterial cells from TNT-mediated cell death and expression and purification analysis of rIFT and rTNT proteins*.* IFT and TNT genes were codon-optimized for expression in *E. coli* and cloned in pET28a and pMTSA expression vectors respectively. For expression, *E. coli* BL21 cells containing IFT-pET28a were induced with 1 mM IPTG for 10 hours at 25˚C and the expressed protein purified from the soluble fraction. TNT-pMTSA transformed *E. coli* C43 cells were induced with 0.2% arabinose at 25˚C for 10 hours and the expressed TNT protein purified from inclusion bodies using Ni-NTA chromatograpy. TNT protein was refolded by gradual removal of urea from the buffer. **(a)** For co-expression of IFT and TNT proteins, *E.coli* BL21 cells were co-transformed with IFT-pET28a and TNT-pMTSA constructs. Confirmation of positive clones was carried out by PCR. **(b)** Schematic representation of the effect of NAD^+^ modulation on bacterial propagation. **(c)** SDS-PAGE analysis of purified and dialysed IFT and TNT proteins detected protein bands of molecular weight ~21.6 kDa and ~24.6 kDa respectively. **(d)** Scheme of the purification of IFT-TNT complex. **(e)** Localization study of co-expression of IFT and TNT proteins. **(f)** Cloned cells were co-induced with 0.2% arabinose and 1 mM IPTG for 10 hours at 25˚C and co-expressed IFT and TNT proteins were purified from the soluble fraction using Ni-NTA chromatography. Co-purified proteins were dialysed and heated at 55˚C. TNT was extracted from the soluble fraction while IFT remained in the insoluble fraction. SDS-PAGE analysis of isolated TNT from heated supernatant of IFT-TNT complex, and IFT from insoluble fraction.

**Supplementary Fig 3. (a)** Far-Western blot analysis confirming the interaction between recombinant TNT and IFT proteins. ETHE1 protein was used as the negative control. **(b)** Determination of NAD^+^-glycohydrolase activity of rTNT 1 (purified from inclusion bodies), rTNT 2 (purified from co-expression of rIFT + rTNT) and its inhibition through rIFT. 30 nM of rTNT was incubated with 1 µM NAD^+^ in the presence and absence of rIFT and the remaining NAD^+^ content determined using the EnzyFluo NAD/NADH kit.

**Supplementary Fig 4. (a)** RT-PCR analysis of transiently transfected cells. **(b)** Determination of macrophage cell death through flow cytometry analysis by PI staining of macrophages transfected with TNT-pEGFPC1 or IFT-pCMV4_nn_ or both plasmids after 48 hours of post-transfection. **(c)** Intracellular translocation of HMGB1. RAW macrophage cells were transiently transfected with TNT-pEGFPC1 or IFT-pCMV4_nn_ or both plasmids. Western blot analysis of cytoplasmic and nuclear fractions was performed after 48 hours of transfection using anti-HMGB1 antibody to determine levels of HMGB1 protein. β-Actin was used as the internal loading control. **(d)** Nuclear to cytosolic translocation of HMGB1 in *M. tuberculosis* H37Rv infected RAW cells with or without IFT expression was analysed by Western blotting after 48 hours post-infection. β-Actin was used as the internal loading control. (**e)** Schematic representation of effect of NAD^+^ modulation on *M. tuberculosis* survival. Mtb, *M. tuberculosis*.

**Supplementary Fig 5.** Analysis of the eryptotic markers in erythrocytes loaded with rTNT and rIFT. **(a)** Schematic representation of loading of erythrocytes with recombinant proteins. Protein-loaded erythrocytes were analysed for markers of eryptosis after incubation at 37˚C for 48 hours. **(b)** Percentage of loaded erythrocytes demonstrating annexin-V binding. **(c)** Analysis of forward scatter in erythrocytes loaded with rTNT and rIFT. Number of cells with decreased forward scatter in each sample was normalized with control. **(d)** Percentage of DCFDA fluorescence positive erythrocytes loaded with rTNT and rIFT. **(e)** Percentage of loaded erythrocytes positive for Fluo-4AM fluorescence. **(f)** Determination of percent survival of *P. falciparum* in erythrocytes loaded with proteins rIFT, rTNT and both, through flow cytometry. Significance of difference in values was calculated using the unpaired t-test, *P<0.05.

**Supplementary Fig 6. (a)** Synthesis of hybrid small molecules. Determination of inhibitory potential of drugs and compounds against NAD^+^-glycohydrolase activity of TNT. Percent decrease in NAD^+^ content represents NAD^+^-glycohydrolase activity. Percent inhibition in NAD^+^-glycohydrolase activity was calculated relative to control. **(b)** 1 µM APBA was incubated with 30 nM of TNT followed by addition of 5 µM NAD^+^ to initiate the reaction; the remaining NAD^+^ level was measured using enzyme coupling method. **(c, d)** Relative NAD^+^ levels of treated samples compared to control. NAD^+^-glycohydrolase assay of purified rTNT (75 nM) at 200 µM NAD^+^ concentration in presence and absence of drugs or compounds (100 µM) by NADH fluorescence method. **(e)** Percent inhibition of NAD^+^-glycohydrolase activity of TNT in the presence of synthesized NAD^+^ analogues or drugs and compounds. Significance of difference in values was calculated using the unpaired t-test, *P<0.05.

**Supplementary Fig 7.** Determination of growth inhibitory potential of small molecules **8, 9, and 10** against *P. falciparum* growth through flow cytometry by ethidium bromide staining.

**Supplementary Table 1.** DNA sequence of IFT gene and TNT gene

**Supplementary table 2.** Chemical properties of hybrid small molecules

**Supplementary Table 3.** Hydrogen bond analysis of TNT-8, TNT-9, TNT-10

**Supplementary Table 4.** Strains, Plasmids and antibodies

**Supplementary Table 5.** Primer sequences

**Supplementary Methods**

**Production of antibodies against IFT and TNT proteins**

Purified and refolded rTNT protein from inclusion bodies, that doesn’t show NAD^+^-glycohydrolase activity, was used to raise antibody (Supplementary Fig 3b). BALB/c mice were immunized subcutaneously with 50-100µg of rIFT or rTNT protein mixed with Freund’s complete adjuvant (Sigma-Aldrich) followed by three boosters of respective proteins formulated with Freund’s incomplete adjuvant (Sigma-Aldrich). Blood sample was collected after third immunization and antibody titers were determined in serum by Enzyme-Linked Immunosorbent Assay (ELISA).

**Nuclear and cytoplasmic extraction**

To separate the nuclear and cytosolic fractions, treated RAW 264.7 macrophage cells were harvested and resuspended in cytoplasmic extraction buffer (5 mM Tris, pH 7.4, 1.5 mM MgCl_2_, 5 mM KCl, 2 mM EGTA, 1 mM PMSF, 1 mM DTT) and incubated on ice for 15 min with repeated vortexing to lyse cells. After centrifugation obtained supernatant (cytoplasmic fraction) was stored with protease inhibitor cocktail (Roche diagnostics). Remaining pellet was consecutively resuspended in a nuclear extraction buffer (10 mM Tris, pH 7.4, 10 mM NaCl, 5 mM EDTA, 1% Triton X-100, 1 mM PMSF) and supernatant (nuclear fraction) was separated by centrifugation, and then stored after addition of protease inhibitor cocktail. To analyse proteins released into culture supernatant, cells grown in either minimal volume or supernatant was concentrated using centricon centrifugal filter (Merck Millipore) to obtain concentrated samples.

**Western blotting**

Equally loaded protein samples were resolved on SDS-PAGE and transferred onto nitrocellulose membrane (Bio-Rad, USA). To identify proteins, the blots were first incubated with respective primary antibodies, followed by detection with HRP-conjugated secondary antibodies. These blots were developed by chemiluminescence using the Luminol reagent (Clarity™ western ECL substrate, Bio-Rad). GAPDH or Actin was used as loading control.

**RT-PCR analysis of IFT and TNT expression in macrophage**

After treatment, total RNA was prepared from cells using TRIzol reagent (Invitrogen) according to the manufacturer's protocol. cDNA synthesis was conducted using random hexamer primers (RevertAid First Strand cDNA synthesis kit, Thermo). PCR amplification of cDNA was conducted using specific primer sets at annealing temperature of 55–60°C for 30 cycles (Supplementary Table 5). GAPDH was used as internal control to normalize expression. To analyse PCR products, each sample was electrophoresed on a 1% agarose gel and detected under UV light.

**Determination of *P. falciparum* growth through flow cytometry**

rTNT and rIFT loaded erythrocytes were infected with *P. falciparum* schizonts and parasite growth was accessed after 48 hours post-infection. To determine inhibitory potential of small molecules on parasite growth, synchronized *P. falciparum* culture at 1% parasitemia and 2% hematocrit was treated with molecules for 48 hours. After incubation, parasites infected erythrocytes were stained with ethidium bromide (10 μM) for 30 minutes in dark and parasite growth was determined by flow cytometry (BD, LSR Fortessa, Becton Dickinson, USA), and data were analysed using FlowJo software (Tree Star Inc, USA).

**Preparation and Spectral data of all hybrid small molecules (1-14):**

**Preparation of 4-(2-bromoethoxy)-2H-chromen-2-one (1):**

To the stirred solution of 4-hydroxycoumarin (5 g, 130.83 mmol) in DMF (30 mL), K_2_CO_3_ (8.53 61.67 mmol) followed by 1,2-dibromo ethane (4 mL 46.20mmol) were added drop wise and reaction mixture was reflux for 3h. The progress of reaction was monitored by TLC, the starting material was consumed and two major spots are formed. After completion of reaction mixture was cooled to room temperature and ice water added, solid was precipitated out then it was filtered to get solid residue. The solid residue was contain two products the pure desired product **1** was obtained, by normal column chromatography using silica gel (100-200 mesh size) 3:7 EtOAc/Hexane as eluent, as white solid (50%).

**4-(2-bromoethoxy)-2H-chromen-2-one (1):** ^1^H NMR (400 MHz, CDCl_3_):δ 7.89-7.86 (dd, 2H, *J* = 1.6, *J* = 8 Hz), 7.59-7.55 (td, 2H *J* = 1.6, *J* = 7.6 Hz), 5.67 (s, 1H ),4.45 (t, 2H, *J*= 5.6 Hz), 3.76 (t, 2H, *J*= 5.6 Hz):^13^C NMR (100 MHz, CDCl_3_):δ 164.9, 162.5, 153.3, 132.6, 124.1, 123.9, 116.9, 115.3, 90.9, 68.5, 27.6; HRMS (ESI) *m/z*: calcd for C_11_H_9_BrO_3_ [M+H]^+^ 268.9813, found 268.9810.

**Preparation of hybrid molecules 2-13:**

To the stirred solution of 4-hydroxycoumarin (heterocycles) (55 mg, 0.339 mmol) in DMF (4 mL), K_2_CO_3_ (94 mg, 0.679 mmol) was added followed by compound **1** (91 mg, 0.340 mmol) and the reaction mixture was stirred at room temperature for 48 h. The progress of reaction mixture was monitored by the TLC. After completion of reaction, it was quenched with ice water, extracted with EtOAc (3 × 20 mL). The combined organic layer washed with saturated brine solution, dried over anhydrous sodium sulphate and concentrate under reduced pressure to get crude product. The crude product was purified by column chromatography (silica gel 100-200 mesh) using 3:7 to 4:1 EtOAc in hexane to get pure product **2** as orange solid (70%). When the same reaction was carried out under microwave heating condition reaction was completed within 30 minute and product yield was higher (79%).

The similar reaction protocol was adopted for the preparation of all the hybrid small molecules **3-13** reported in this paper. Compound **14** was purchased from Aldrich.

**4,4'-(ethane-1,2-diylbis(oxy))bis(2H-chromen-2-one) (2):** ^1^H NMR (400 MHz, CDCl_3_):δ 7.83-7.80 (dd, 2H *J* = 1.6, *J* =8.0 Hz), 7.596-7.553(m, 2H), 7.36-7.33 (d, 2H *J* = 8.4 Hz), 7.29- 7.27 (m, 2H), 5.81 (s, 2H), 4.62 (s, 4H);^13^C NMR (100 MHz, CDCl_3_):δ 165.1, 162.5, 153.3, 132.7, 124.7, 122.9, 116.8, 115.2, 91.1, 66.7; HRMS (ESI) *m/z*: calculated for C_20_H_14_O_6_ [M+H]^+^ 351.0869, found 351.0861.

**4-(2-(6-amino-9H-purin-9-yl)ethoxy)-2H-chromen-2-one (3):** The crude product was purified by column chromatography (silica gel 100-200 mesh) using 2-3% MeOH in CH_2_Cl_2_ to get pure product as white solid (65%).^1^H NMR (400 MHz, DMSO-*d*6): δ 8.30 (s, 1H),8.18 (s, 1H), 7.75-7.72 (dd, 1H, *J* = 1.2, *J =* 7.6 Hz), 7.65-7.60 (td, 1H, *J* = 1.6, *J* = 7.2 Hz), 7.37-7.30 (m, 2H), 7.09 (brs, 2H), 5.93 (s, 1H ), 4.68-4.66 (t, 2H, *J*= 4.4 Hz), 4.58-4.55 (t, 2H, *J*= 4.8 Hz);^13^C NMR (100 MHz, DMSO-*d*6): δ 167.2, 165.1, 153.2, 141.6, 133.3, 124.59, 123.3, 116.8, 91.4, 68.3, 42.3; HRMS (ESI) *m/z*: calculated for C_16_H_13_N_5_O_3_ [M+H]^+^ 324.1097, found 324.1099.

**1-(2-((2-oxo-2H-chromen-4-yl)oxy)ethyl)indoline-2,3-dione (4):** The crude product was purified by column chromatography (silica gel 100-200 mesh) using 60-80% EtOAc in hexane to get pure product as orange solid (78%).^1^H NMR (400 MHz, DMSO-*d*6):δ 7.75-7.71 (t, 1H, *J* = 7.6 Hz), 7.65-7.60 (m, 2H), 7.56-7.54 (d, 1H, *J* = 7.2 Hz), 7.41-7.34 (dd, 2H, *J* = 8.0 Hz, *J*= 1.8 Hz), 7.32-7.28 (t, 1H, *J* = 7.6 Hz), 7.17-7.13 (t, 1H, *J* = 7.6 Hz), 5.88 (s, 1H),4.47-4.45 (t, 2H, *J*= 4.8 Hz), 4.24-4.22 (t, 2H, *J*= 4.8 Hz);^13^C NMR (100 MHz, DMSO-*d*6): δ 183.5, 165.2, 161.9, 158.8, 153.1, 151.1, 138.5, 133.2, 124.9, 124.4, 123.7, 123.43, 118.0, 116.8,115.5, 111.5, 91.3, 67.4, 39.1; HRMS (ESI) *m/z*: calculated for C_19_H_13_NO_5_ [M+H]^+^ 336.0872, found 336.0870.

**4-(2-(1H-benzo[d]imidazol-1-yl)ethoxy)-2H-chromen-2-one (5):** ^1^H NMR (400 MHz, DMSO-*d*6) δ 8.41 (s, 1H ), 7.76-7.74 (d, 1H, *J* = 8.0 Hz), 7.69-7.60 (m, 3H), 7.36-7.28 (m, 3H), 7.24-7.20 (t, 1H, *J* = 8,Hz), 5.90 (s, 1H), 4.83-4.80 (t, 2H, *J*= 4.8 Hz), 4.59-4.57 (t, 2H, *J*= 4.8 Hz);^13^C NMR (100 MHz, DMSO-*d*6):δ 164.8, 161.8, 153.1, 144.9, 143.8, 134.3, 133.25, 124.5, 123.2, 122.8, 122.0, 119.9, 116.8, 115.3, 110.9, 91.5, 68.5, 43.4; HRMS (ESI) *m/z*: calculated for C_18_H_14_N_2_O_3_ [M+H]^+^ 307.1083, found 307.1081.

**3-(2-((2-oxo-2H-chromen-4-yl)oxy)ethyl)quinazolin-4(3H)-one (6):** ^1^H NMR (400 MHz, DMSO-*d*6) δ 8.59 (s, 1H), 8.20-8.17 (dd, 1H *J* = 1.2, *J* = 8.0 Hz), 7.83-7.80 (m, 2H), 7.69-7.55 (m, 2H), 7.38-7.38 (t, 1H, *J* = 1.6 Hz), 7.37-7.35 (m, 2H), 5.95 (s, 1H), 4.53-4.51 (t, 4H, *J*= 3.6 Hz),^13^C NMR (100 MHz, DMSO-*d*6) δ 164.8, 161.8, 160.7, 153.2, 148.6, 148.3,134.8, 127.6, 127.5, 126.5, 124.6, 123.2, 116.8, 115.5, 91.5, 67.5, 45.2; HRMS (ESI) *m/z*: calculated for C_19_H_14_N_2_O_4_ [M+H]^+^ 335.1032, found 335.1036.

**4-(2-(4H-1,2,4-triazol-4-yl)ethoxy)-2H-chromen-2-one (7):** ^1^H NMR (400 MHz, CDCl_3_): δ 8.41 (s, 2H), 7.98 (s, 1H), 7.63-7.61 (d, 2H, *J* = 8.0 Hz), 7.55-7.51 (t, 1H,*J* = 8.4Hz), 5.66 (s, 1H), 4.70-4.68 (t, 2H, *J*= 4.8 Hz), 4.52-4.50 (t, 2H, *J*= 5.2 Hz);^13^C NMR (100 MHz, CDCl_3_): δ 164.5, 162.1, 153.3, 132.7, 124.1, 122.5, 116.9, 115.0, 91.2, 66.4, 48.2; HRMS (ESI) *m/z*: calculated for C_13_H_11_N_3_O_3_ [M+H]^+^ 258.0879, found 258.0871.

**4-(2-((2-oxo-2H-chromen-4-yl)oxy)ethoxy)quinolin-2(1H)-one (8):** ^1^H NMR (400 MHz, DMSO-*d*6): δ 8.12 (m, 1H), 7.83-7.80 (dd, 1H *J* = 1.6, *J* =8.0 Hz), 7.596-7.553 (m, 2H), 7.36-7.33 (m, 2H), 7.29- 7.27 (m, 2H), 5.90 (s, 1H), 5.81 (s, 1H), 4.62 (s, 4H); ^13^C NMR (100 MHz, DMSO-*d*6): δ 168.1, 165.1, 163.1, 162.5, 153.3, 152.5, 132.7, 132.2, 124.7, 124.5, 122.9, 122.7, 117.1, 116.8, 115.2, 115.1, 91.1, 66.7, 65.6;HRMS (ESI) *m/z*: calculated for C_20_H_15_NO_5_ [M+H]^+^ 350.1028, found 350.1025.

**5-bromo-1-(2-((2-oxo-2H-chromen-4-yl)oxy)ethyl)indoline-2,3-dione (9):** ^1^H NMR (400 MHz, DMSO-*d*6): δ 8.12 (s, 1H), 7.90 (d, 1H, *J* = 8.0 Hz), 7.75-7.71 (d, 1H, *J* = 7.6,Hz), 7.56-7.54 (d, 1H, *J* = 7.2 Hz), 7.41-7.34 (dd, 1H, *J* = 8.0 Hz, *J* = 1.8 Hz), 7.32-7.28 (t, 1H, *J* = 7.6 Hz), 7.17-7.13 (t, 1H, *J* = 7.6 Hz), 5.88 (s, 1H),4.47-4.45 (t, 2H, *J*= 4.8 Hz), 4.24-4.22 (t, 2H, *J*= 4.8 Hz); ^13^C NMR (100 MHz, DMSO-*d*6): δ 183.4, 165.1, 162.0, 158.7, 153.0, 151.2, 138.5, 133.2, 124.9, 124.4, 123.7, 123.4, 119.2, 118.0, 116.8,115.5, 111.5, 91.3, 67.5, 39.2; HRMS (ESI) *m/z*: calculated forC_19_H_12_BrNO_5_ [M+H]^+^ 413.9977, found 413.9972.

**4-(2-(6-chloro-2-fluoro-9H-purin-9-yl)ethoxy)-2H-chromen-2-one (10):** ^1^H NMR (400 MHz, DMSO-*d*6): δ 8.30 (s, 1H),7.76-7.73 (dd, 1H, *J* = 1.5, *J =*8.0 Hz), 7.66-7.61 (td, 1H, *J* = 1.6, *J* = 8.0 Hz), 7.38-7.31 (m, 2H), 5.92 (s, 1H ), 4.67-4.65 (t, 2H, *J*= 4.4 Hz), 4.57-4.55 (t, 2H, *J*= 4.8 Hz);^13^C NMR (100 MHz, DMSO-*d*6): δ 169.2, 165.1, 161.3, 158.2, 153.2, 141.6, 133.3, 124.59, 123.3, 116.8, 91.4, 68.3, 42.3;HRMS (ESI) *m/z*: calculated for C_16_H_10_ClFN_4_O_3_ [M+H]^+^ 361.0504, found 361.0501.

**4-(2-(pyrrolidin-1-yl)ethoxy)-2H-chromen-2-one (11):** ^1^H NMR (400 MHz, DMSO-*d*6) δ 7.77-7.75 (dd, 1H *J* = 1.6 Hz, *J* = 8.0 Hz), 7.66-7.62 (td, 1H, *J* = 1.6 Hz, *J* = 8.0 Hz), 7.39-7.34 (m, 2H), 5.91 (s, 1H), 4.31-4.28 (t, 2H, *J*= 7.5 Hz), 2.80-2.78 (t, 2H, *J* = 7.5 Hz), 2.51-2.49 (m, 4H), 1.67-1.65 (m, 4H); ^13^C NMR (100 MHz, DMSO-*d*6): δ 165.4, 162.1, 153.3, 133.2, 124.6, 123.1, 117.0, 115.7, 91.2, 68.2, 57.0, 54.5, 24.1; HRMS (ESI) *m/z*: calculated for C_15_H_17_NO_3_ [M+H]^+^ 260.1287, found 260.1282.

**4-(2-(piperidin-1-yl)ethoxy)-2H-chromen-2-one (12):** ^1^H NMR (400 MHz, DMSO-*d*6) δ 7.79-7.76 (dd, 1H *J* = 1.6 Hz, *J* = 8 Hz), 7.67-7.63(td, 1H, *J* = 1.6 Hz, *J* = 8 Hz), 7.40-7.35 (m, 2H), 5.92 (s, 1H), 4.32-4.29 (t, 2H, *J*= 5.6 Hz), 2.80-2.78(t, 2H, *J* = 5.2 Hz), 2.45-2.42 (m, 4H), 1.52-1.47(m, 4H), 1.40-1.35 (m, 2H); ^13^C NMR (100 MHz, DMSO-*d*6): δ 165.3, 162.0, 153.2, 133.1, 124.6, 123.2, 116.9, 115.7, 91.1, 68.1, 57.0, 54.6, 26.0, 24.2; HRMS (ESI) *m/z*: calculated forC_16_H_19_NO_3_ [M+H]^+^ 274.1443, found 274.1440.

**4-(2-morpholinoethoxy)-2H-chromen-2-one (13):** ^1^H NMR (400 MHz, DMSO-*d*6) δ 7.80-7.77 (dd, 1H, *J* = 1.6, *J* = 8.0 Hz), 7.68-7.63(m, 1H), 7.40-7.35 (m, 2H), 5.93 (s, 1H), 4.34-4.32 (t, 2H, *J*= 5.6 Hz), 3.59-3.57(t, 4H, *J* = 4.4 Hz), 2.84-2.81(t, 2H, *J* = 5.2 Hz); ^13^C NMR (100 MHz, DMSO-*d*6) δ 165.3, 162.1, 153.2, 133.2, 124.7, 123.3, 116.9, 115.7, 91.2, 67.9, 66.6, 56.6, 53.8; HRMS (ESI) *m/z*: calculated for C_15_H_17_NO_4_ [M+H]^+^ 276.1236, found 276.1231.

**Quinazolin-4(3H)-one (14):** ^1^H NMR (400 MHz, DMSO-*d*6) δ 12.31 (brs, 1H), 8.17 (dd, 1H, 8.0 Hz, 1.6 Hz), 8.15 (s, 1H), 7.84 (td, 1H, 8.0 Hz, 1.5 Hz). 7.70 (dd, 1H, 8.0 Hz, 1.6 Hz), 7.55 (td, 1H, 8.0 Hz, 1.5 Hz); ^13^C NMR (100 MHz, DMSO-*d*6) δ 160.7, 148.7, 145.3, 134.2, 127.1, 126.6, 125.8, 122.6; HRMS (ESI) *m/z*: calculated for C_8_H_6_N_2_O [M+H]^+^ 147.0558, found 147.0555.

**References**

1. Walliker, D. *et al.* Genetic analysis of the human malaria parasite Plasmodium falciparum. *Science* **236**, 1661-1666 (1987).
2. Cole, S. T. *et al.* Deciphering the biology of Mycobacterium tuberculosis from the complete genome sequence. *Nature* **393**, 537-544 (1998).
3. Kumar, K. *et al.* Phenylalanine-rich peptides potently bind ESAT6, a virulence determinant of Mycobacterium tuberculosis, and concurrently affect the pathogen's growth. *PLoS One* **4**, e7615 (2009).
4. Ghosh, A. *et al.* Expression of the ARPC4 subunit of human Arp2/3 severely affects mycobacterium tuberculosis growth and suppresses immunogenic response in murine macrophages. *PLoS One* **8**, e69949 (2013).
5. Tharad, M. *et al.* A three-hybrid system to probe in vivo protein-protein interactions: application to the essential proteins of the RD1 complex of M. tuberculosis. *PLoS One* **6**, e27503 (2011).
