# Supplementary material for: Pathogen induced subversion of NAD+ metabolism mediating host cell death: a target for development of chemotherapeutics
Source: Cell Death Discov. 2021 Jan 13;7:10. doi: 10.1038/s41420-020-00366-z (PMC7806871; doi:10.1038/s41420-020-00366-z)
Supplement: Supplementary file 2 — Supplementary table 1 [file 41420_2020_366_MOESM2_ESM.docx]

**Supplementary Table 1. DNA sequence of IFT gene and TNT gene**

| Gene Name | Gene Sequence | Length |
| --- | --- | --- |
| IFT (Codon optimized) | ATGACCATCGGCGTGGACCTGAGCACCGACCTGCAAGACTGGATTCGTCTGAGCGGTATGAACATGATTCAAGGCAGCGAAACCAATGACGGTCGTACCATCCTGTGGAACAAGGGTGGCGAGGTTCGTTATTTCATCGACCGTCTGGCGGGTTGGTACGTGATTACCAGCAGCGATCGTATGAGCCGTGAGGGCTATGAGTTCGCTGCGGCGAGCATGAGCGTTATTGAGAAATACCTGTATGGTTACTTTGGTGGCAGCGTGCGTAGCGAGCGTGAACTGCCGGCGATCCGTGCGCCGTTCCAGCCGGAGGAACTGATGCCGGAATATAGCATTGGCACCATGACCTTTGCGGGCCGTCAACGTGACACCCTGATCGATAGCAGCGGCACCGTGGTTGCGATTACCGCGGCGGACCGTCTGGTTGAACTGAGCCACTACCTGGATGTGAGCGTTAACGTGATCAAGGATAGCTTTCTGGATAGCGAAGGCAAACCGCTGTTCACCCTGTGGAAGGATTACAAGGGC | 540 |
| TNT (Codon optimized) | GGTTGGCACCGTCTGAGCGATGAGGCGGTTGATCCGCAATATGGCGAGCCGCTGAGCCGTCACTGGGACTTTACCGACAATCCGGCGGACCGTAGCCGTATCAACCCGGTGGTTGCGCAACTGATGGAGGACCCGAACGCGCCGTTTGGTCGTGATCCGCAGGGTCAACCGTACACCCAGGAGCGTTATCAAGAACGTTTCAACAGCGTGGGTCCGTGGGGCCAGCAATACAGCAACTTTCCGCCGAACAACGGTGCGGTTCCGGGCACCCGTATTGCGTACACCAACCTGGAAAAGTTTCTGAGCGATTATGGTCCGCAGCTGGACCGTATCGGTGGCGATCAAGGCAAATATCTGGCGATTATGGAACATGGCCGTCCGGCGAGCTGGGAACAACGTGCGCTGCATGTGACCAGCCTGCGTGACCCGTACCACGCGTATACCATCGATTGGCTGCCGGAGGGCTGGTTCATTGAGGTGAGCGAAGTTGCGCCGGGTTGCGGTCAGCCGGGTGGCAGCATCCAAGTTCGTATTTTTGACCACCAAAATGAGATGCGTAAAGTTGAGGAGCTGATTCGTCGCGGCGTGCTGCGTCAG | 609 |
