# Supplementary material for: Pathogen induced subversion of NAD+ metabolism mediating host cell death: a target for development of chemotherapeutics
Source: Cell Death Discov. 2021 Jan 13;7:10. doi: 10.1038/s41420-020-00366-z (PMC7806871; doi:10.1038/s41420-020-00366-z)
Supplement: Supplementary file 3 — Supplementary table 2 [file 41420_2020_366_MOESM3_ESM.docx]

**Supplementary Table 2. Chemical properties of hybrid small molecules**

| Compounds | Compound Structure | Molecular Formula | Molecular  Weight | Remaining  NAD^+^ % | Percent  Inhibition |
| --- | --- | --- | --- | --- | --- |
| 1 |  | C_11_H_9_BrO_3_ | 267.97 | 70.83 | 66.53 |
| 2 |  | C_20_H_14_O_6_ | 350.07 | 33.30 | 23.43 |
| 3 |  | C_16_H_13_N_5_O_3_ | 323.10 | 77.47 | 74.14 |
| 4 |  | C_19_H_13_NO_5_ | 335.07 | 49.50 | 42.04 |
| 5 |  | C_18_H_14_N_2_O_3_ | 306.10 | 62.59 | 57.07 |
| 6 |  | C_19_H_14_N_2_O_4_ | 334.09 | 17.96 | 5.85 |
| 7 |  | C_13_H_11_N_3_O_3_ | 257.08 | 17.38 | 5.16 |
| 8 |  | C_20_H_15_NO_5_ | 349.09 | 95.02 | 94.28 |
| 9 |  | C_19_H_12_BrNO_5_ | 412.98 | 94.53 | 93.71 |
| 10 |  | C_16_H_10_ClFN_4_O_3_ | 360.04 | 92.88 | 91.82 |
| 11 |  | C_15_H_17_NO_3_ | 259.12 | 25.84 | 14.86 |
| 12 |  | C_16_H_19_NO_3_ | 273.13 | 22.73 | 11.30 |
| 13 |  | C_15_H_17_NO_4_ | 275.11 | 16.79 | 4.49 |
| 14 |  | C_8_H_6_N_2_O | 146.04 | 20.92 | 9.23 |
| Without inhibitor | - | - | - | 12.88 | - |
