# Supplementary material for: Pathogen induced subversion of NAD+ metabolism mediating host cell death: a target for development of chemotherapeutics
Source: Cell Death Discov. 2021 Jan 13;7:10. doi: 10.1038/s41420-020-00366-z (PMC7806871; doi:10.1038/s41420-020-00366-z)
Supplement: Supplementary file 4 — Supplementary table 3 [file 41420_2020_366_MOESM4_ESM.docx]

**Supplementary Table 3. Hydrogen bond analysis of TNT-8, TNT-9, TNT-10**

| **TNT-8 COMPLEX** | | | | | | | |
| --- | --- | --- | --- | --- | --- | --- | --- |
| **Index** | **Residue** | **AA** | **Distance H-A** | **Distance D-A** | **Donor Angle** | **Donor Atom** | **Acceptor Atom** |
| **1** | **731B** | **ASN** | **2.33** | **3.02** | **138.13** | **861 [Nam]** | **2083 [O2]** |
| **2** | **764B** | **LYS** | **2.06** | **2.85** | **156.34** | **1193 [N3]** | **2098 [O2]** |
| **3** | **764B** | **LYS** | **2.52** | **3.02** | **110.1** | **1192 [N3]** | **2102 [O2]** |
| **4** | **765B** | **TYR** | **3.29** | **3.97** | **137.27** | **1218 [N3]** | **2102 [O2]** |
| **5** | **822B** | **GLN** | **3.47** | **3.93** | **116.17** | **1801 [Nam]** | **2082** [**^1^**](#_ENREF_1) |
| **6** | **822B** | **GLN** | **1.65** | **2.63** | **165.83** | **2082** [**^1^**](#_ENREF_1) | **1800 [O2]** |
| **7** | **824B** | **ARG** | **3.55** | **4.07** | **121.99** | **1822 [Ng+]** | **2083 [O2]** |
|  |  |  |  |  |  |  |  |

| **TNT-9 COMPLEX** | | | | | | | |
| --- | --- | --- | --- | --- | --- | --- | --- |
| **Index** | **Residue** | **AA** | **Distance H-A** | **Distance D-A** | **Donor Angle** | **Donor Atom** | **Acceptor Atom** |
| **1** | **765B** | **TYR** | **1.86** | **2.69** | **167.32** | **1166 [O3]** | **1995 [O3]** |
| **TNT-10 COMPLEX** | | | | | | | |
| **Index** | **Residue** | **AA** | **Distance H-A** | **Distance D-A** | **Donor Angle** | **Donor Atom** | **Acceptor Atom** |
| **1** | **764B** | **LYS** | **2.1** | **2.9** | **153.46** | **1142 [Nam]** | **1992 [O2]** |
