# Supplementary material for: Pathogen induced subversion of NAD+ metabolism mediating host cell death: a target for development of chemotherapeutics
Source: Cell Death Discov. 2021 Jan 13;7:10. doi: 10.1038/s41420-020-00366-z (PMC7806871; doi:10.1038/s41420-020-00366-z)
Supplement: Supplementary file 5 — Supplementary table 4 [file 41420_2020_366_MOESM5_ESM.docx]

| **Strains** | **Source/References** |
| --- | --- |
| *E. coli* BL21 (DE3)  *P. falciparum* 3D7  *M. tuberculosis* H37Rv  *M. tuberculosis* H37Rv-GFP | Novagen, USA  Malaria Research and Reference Reagent Resource Center (MR4) Chloroquine sensitive strain^1^  ^2^  ^3^ |
| **Plasmids** |  |
| pET28a  pMTSA  pFLAGCMV_4_ (minor modification)  pEGFPC1 | [^4^](#_ENREF_4)  [^5^](#_ENREF_5)  Sigma Aldrich, USA  Addgene, USA |
| **Cell lines** |  |
| RAW 264.7 | ATCC |
| **Antibodies** |  |
| Anti-HMGB1 pAb (rabbit)  Anti-IFT pAb (mouse)  Anti-TNT pAb (mouse)  Alexa Fluor 594 secondary antibody  HRP-conjugated secondary antibody | Invitrogen (PA1-16926)  In house  In house  Thermo Scientific, USA  Thermo Scientific, USA |
| **Drugs or Compounds**  3-Aminophenylboronic acid (APBA)  Ribavirin  Pyrazinamide  Nicotinamide | Sigma (287512)  Sigma (R9644)  Sigma (P7136)  Cayman chemicals (11127) |

**Supplementary Table 4. Strains, Plasmids, Antibodies**
