# Supplementary material for: Pathogen induced subversion of NAD+ metabolism mediating host cell death: a target for development of chemotherapeutics
Source: Cell Death Discov. 2021 Jan 13;7:10. doi: 10.1038/s41420-020-00366-z (PMC7806871; doi:10.1038/s41420-020-00366-z)
Supplement: Supplementary file 6 — Supplementary table 5 [file 41420_2020_366_MOESM6_ESM.docx]

**Supplementary Table 5. Primer sequences**

| **Primers** | **Sequence** |
| --- | --- |
| IFT-For  IFT-Rev  TNT-For  TNT-Rev  TNT-For  (cloning in EGFPC1) | 5’-CAGAATTCTACGTAATGACCATCGGCGTGGACCT-3’  5’-CCGAATTCTACGTAGCCCTTGTAATCCTTCCACAGG-3’  5’-CCGAATTCTACGTAGGTTGGCACCGTCTGAGCGA-3’  5’-GAGAATTCTACGTACTGACGCAGCACGCCGCGAC-3’  5’-CCGAATTCTACGTACGGTTGGCACCGTCTGAGCGA-3’ |
