# Supplementary figures and images for: Pathogen induced subversion of NAD+ metabolism mediating host cell death: a target for development of chemotherapeutics
Source: Cell Death Discov. 2021 Jan 13;7:10. doi: 10.1038/s41420-020-00366-z (PMC7806871; doi:10.1038/s41420-020-00366-z)

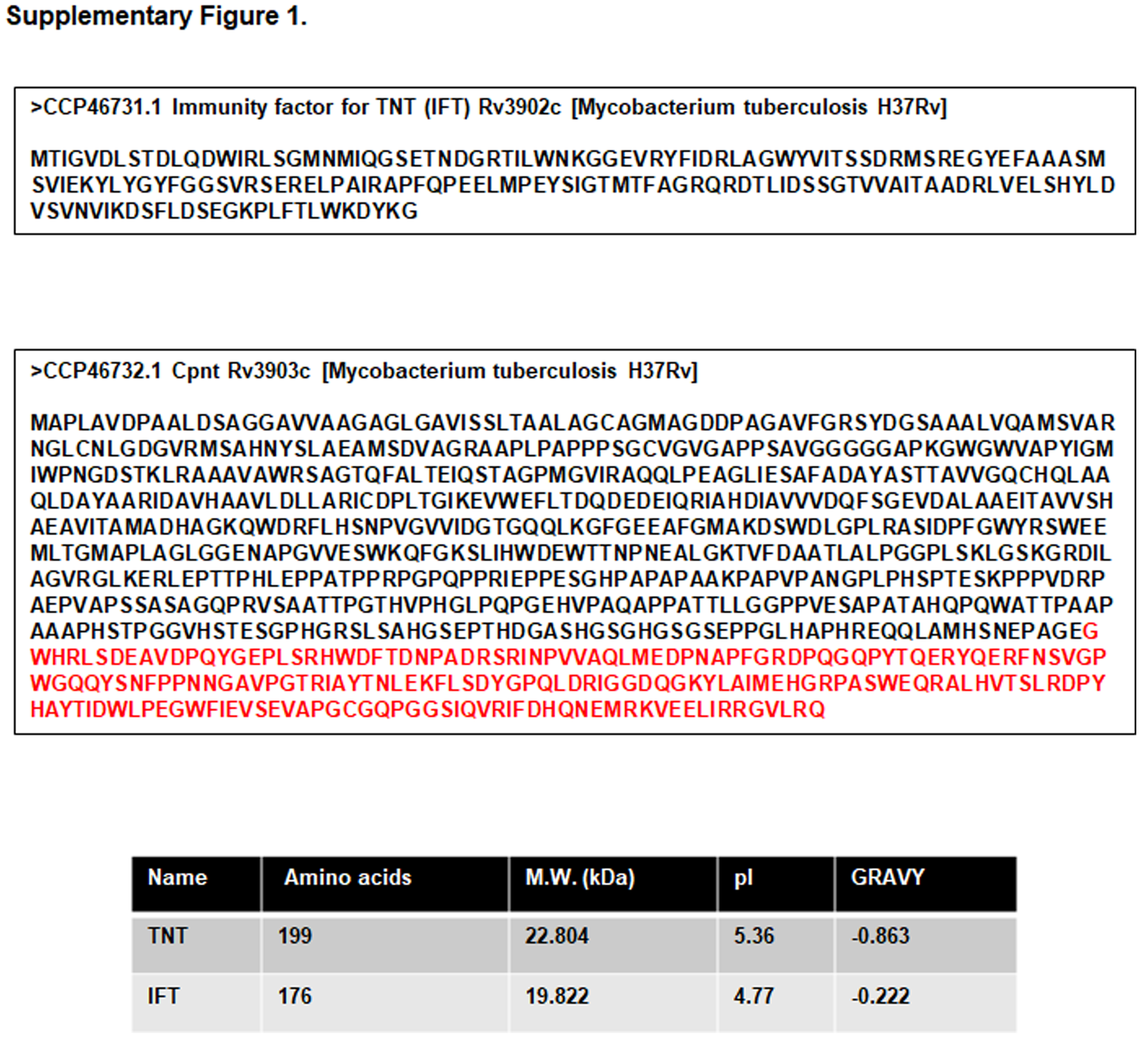

Supplement: Supplementary file 7 — Supplementary Figure 1 [file 41420_2020_366_MOESM7_ESM.tif]

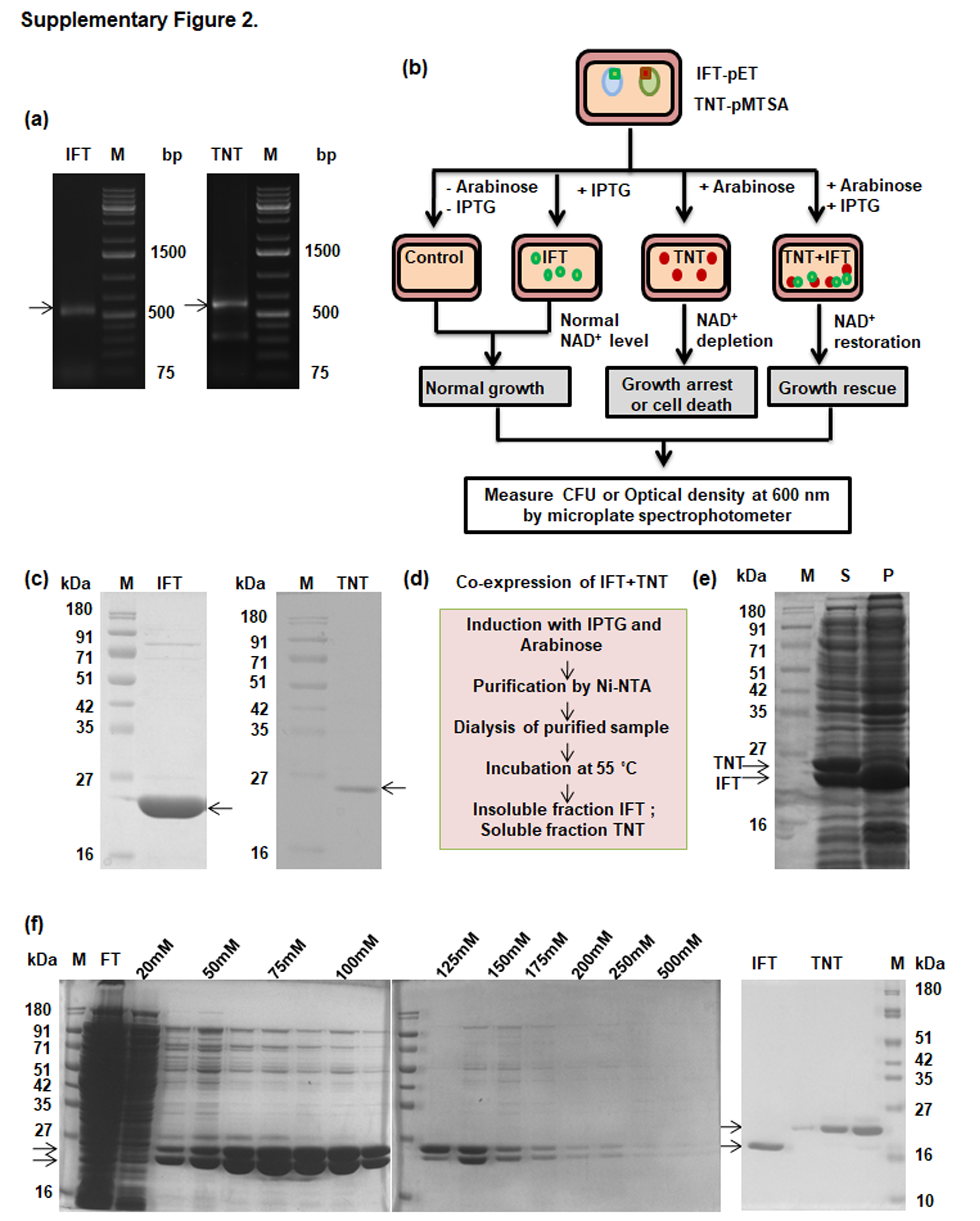

Supplement: Supplementary file 8 — Supplementary Figure 2 [file 41420_2020_366_MOESM8_ESM.tif]

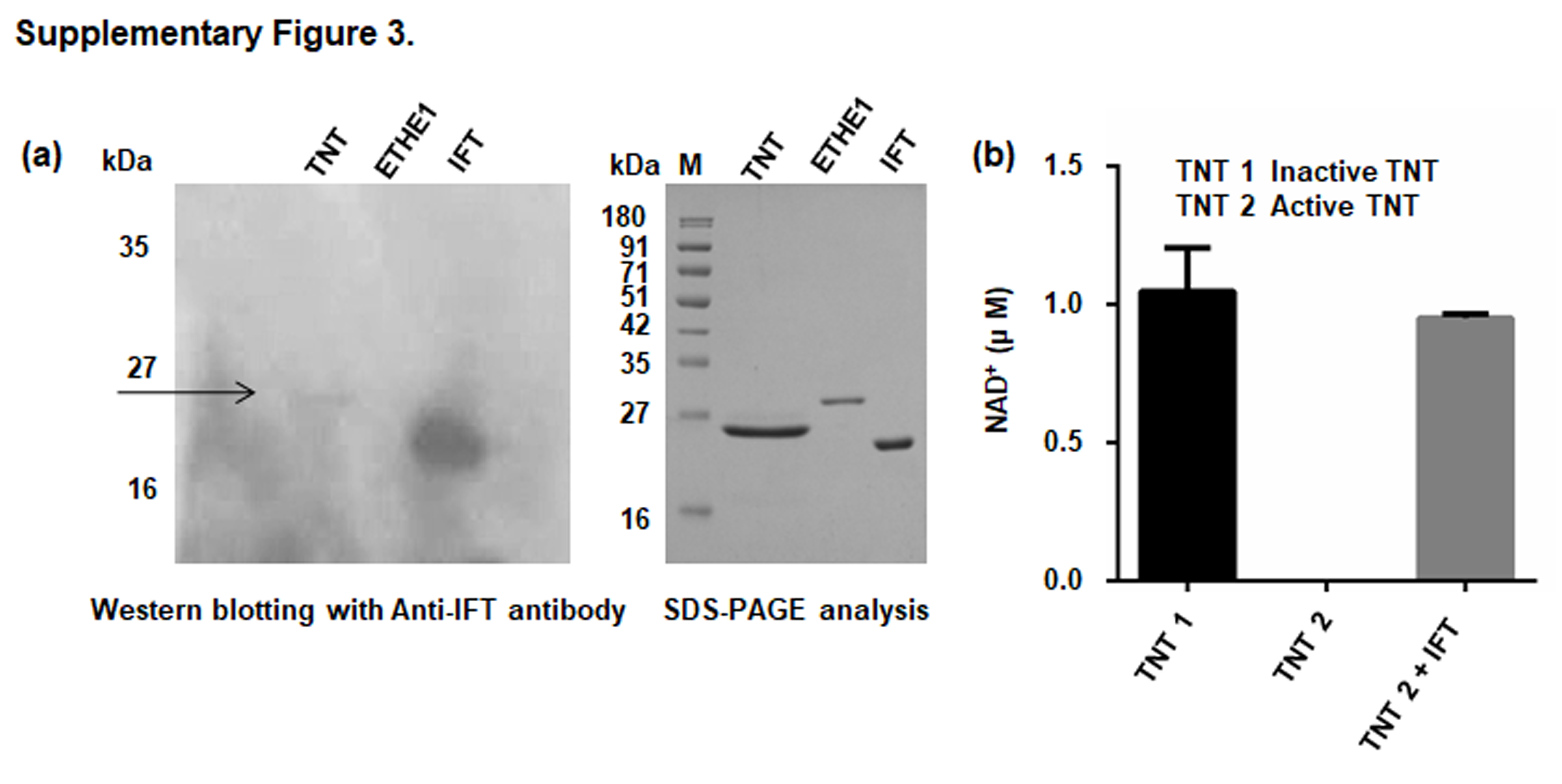

Supplement: Supplementary file 9 — Supplementary Figure 3 [file 41420_2020_366_MOESM9_ESM.tif]

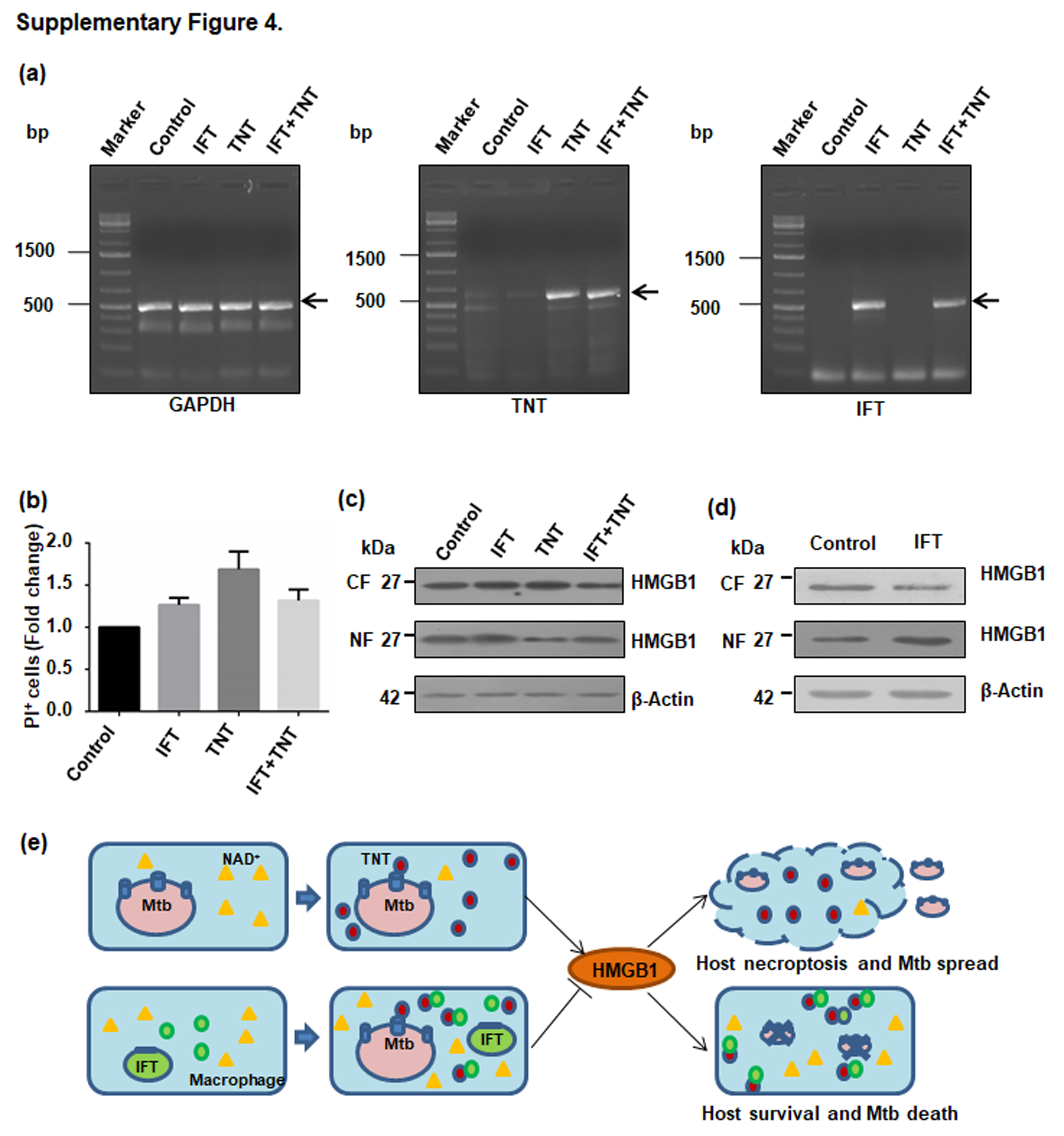

Supplement: Supplementary file 10 — Supplementary Figure 4 [file 41420_2020_366_MOESM10_ESM.tif]

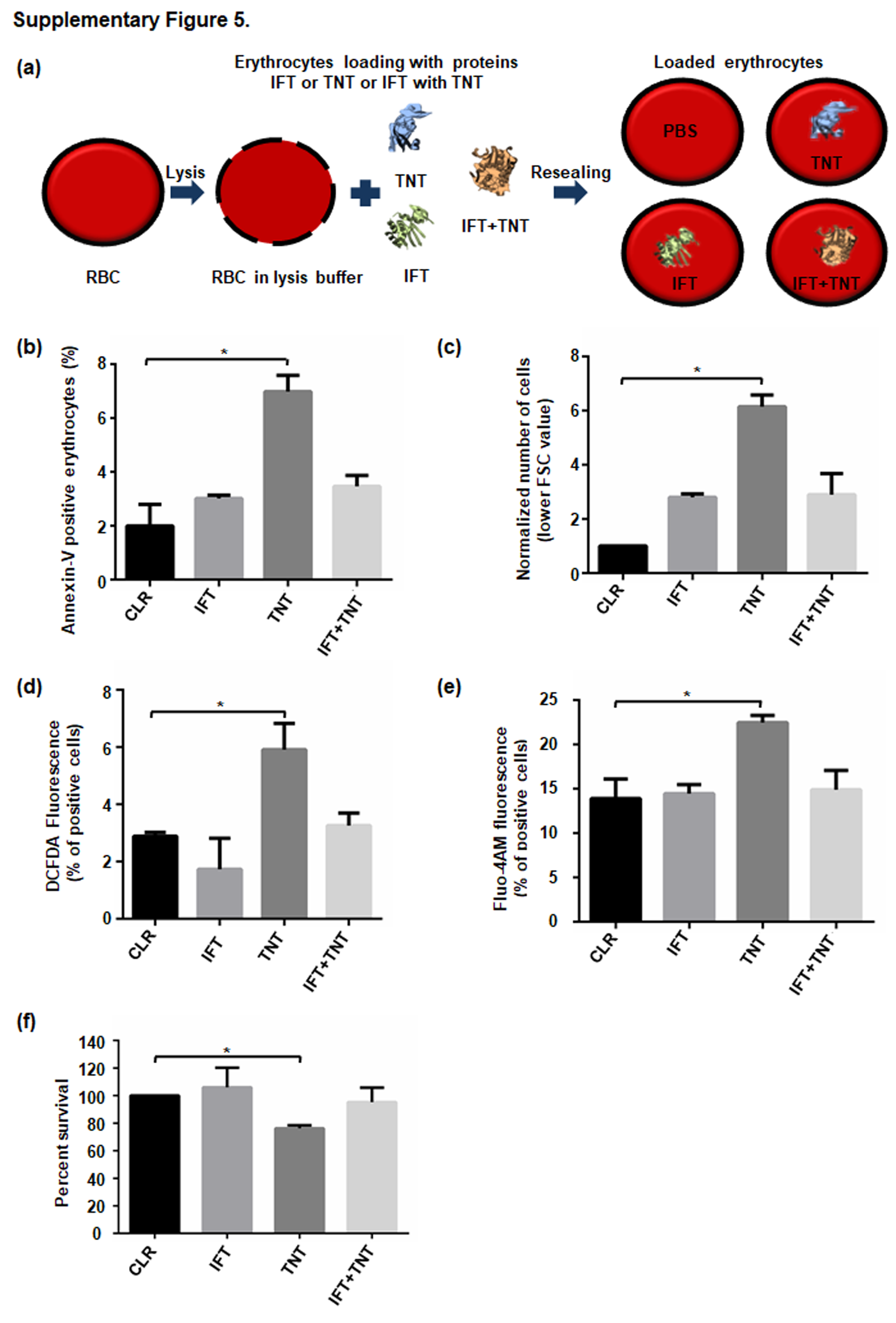

Supplement: Supplementary file 11 — Supplementary Figure 5 [file 41420_2020_366_MOESM11_ESM.tif]

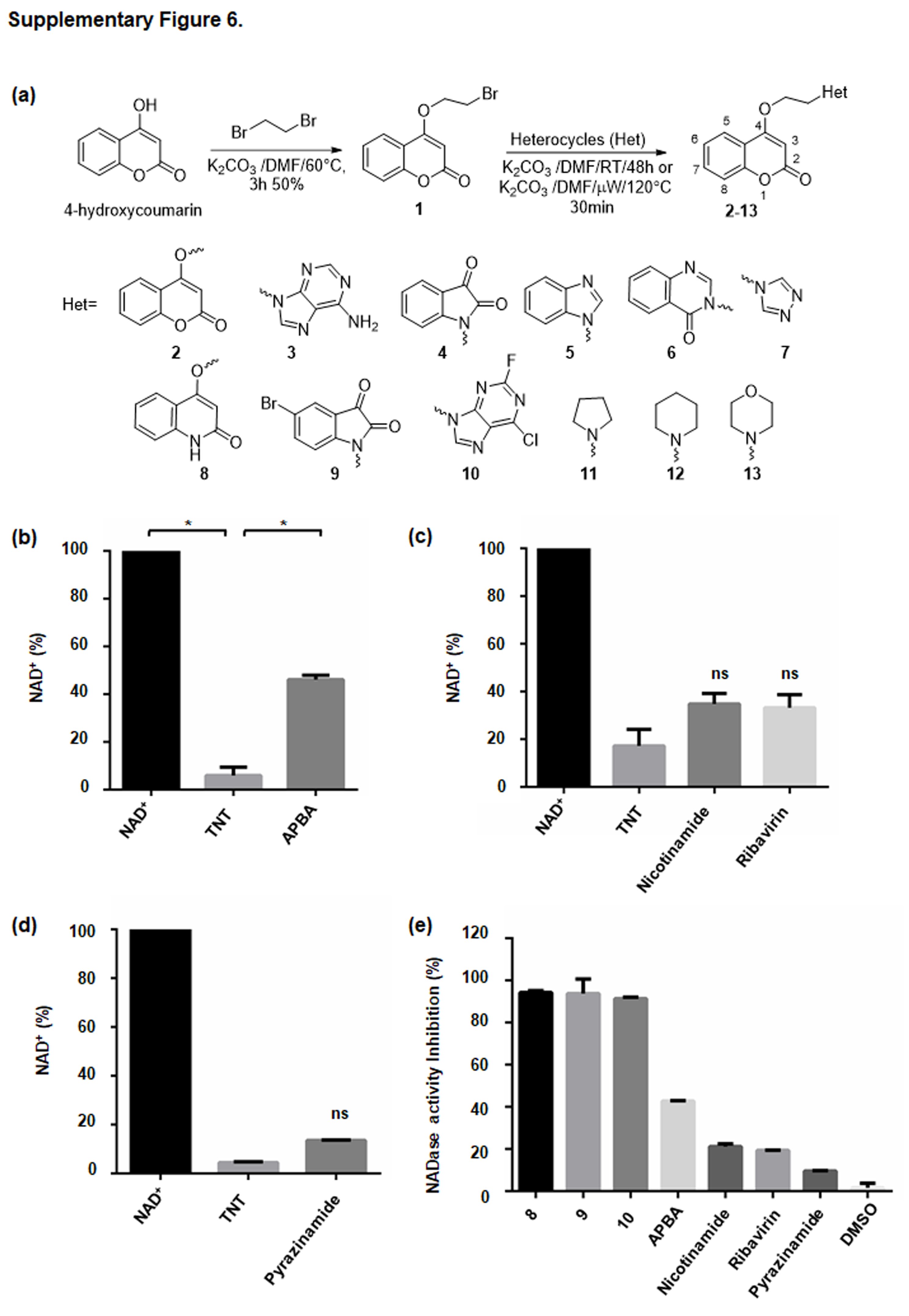

Supplement: Supplementary file 12 — Supplementary Figure 6 [file 41420_2020_366_MOESM12_ESM.tif]

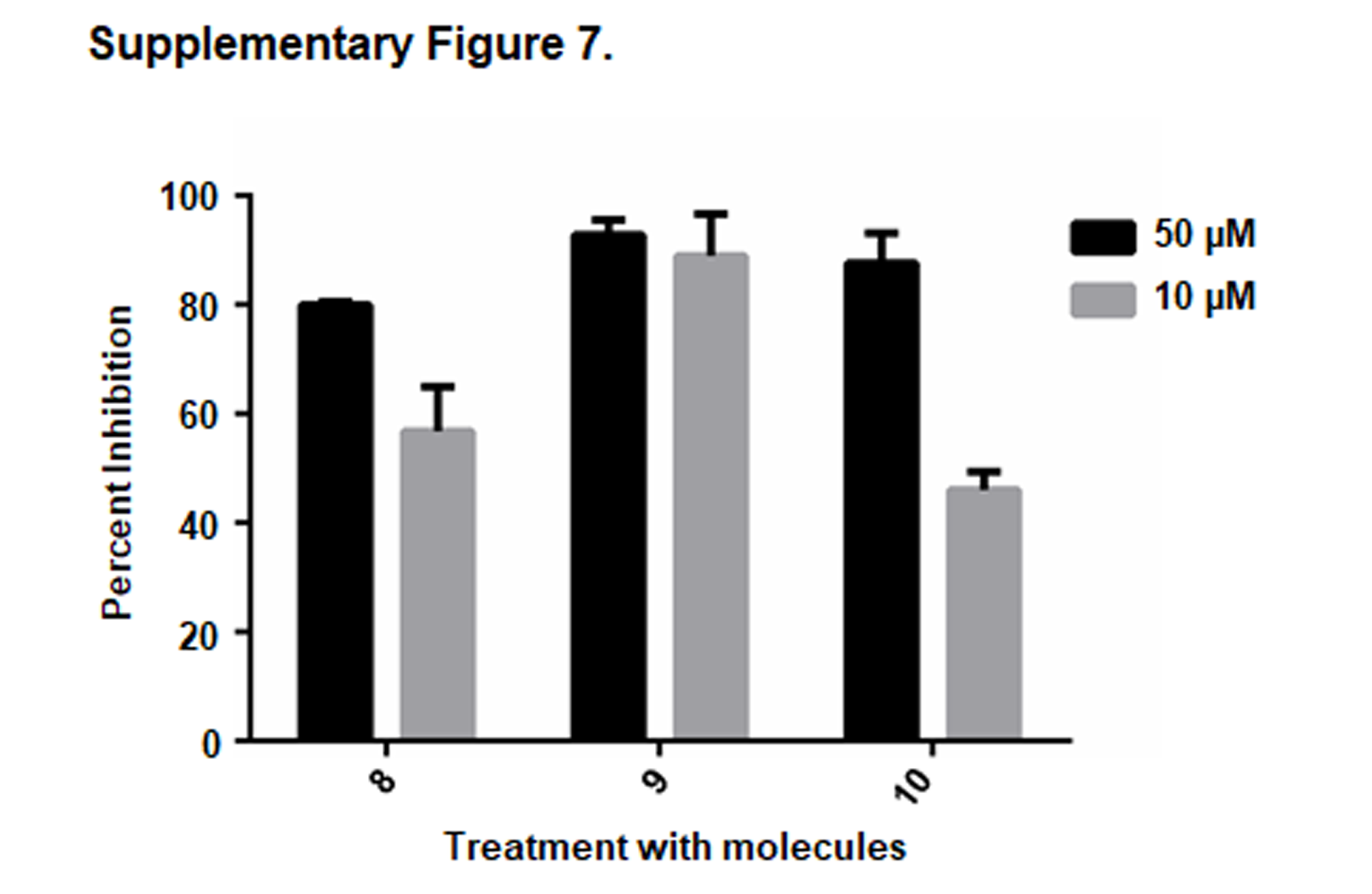

Supplement: Supplementary file 13 — Supplementary Figure 7 [file 41420_2020_366_MOESM13_ESM.tif]
